# A laterally-fused N-heterocyclic carbene framework from polysubstituted aminoimidazo[5,1-*b*]oxazol-6-ium salts

**DOI:** 10.3762/bjoc.20.54

**Published:** 2024-03-18

**Authors:** Andrew D Gillie, Matthew G Wakeling, Bethan L Greene, Louise Male, Paul W Davies

**Affiliations:** 1 School of Chemistry, University of Birmingham, Birmingham, B15 2TT, UK

**Keywords:** annulation, carbene, gold, imidazolium, NHC

## Abstract

A polysubstituted 3-aminoimidazo[5,1-*b*]oxazol-6-ium framework has been accessed from a new nitrenoid reagent by a two-step ynamide annulation and imidazolium ring-formation sequence. Metalation with Au(I), Cu(I) and Ir(I) at the C2 position provides an L-shaped NHC ligand scaffold that has been validated in gold-catalysed alkyne hydration and arylative cyclisation reactions.

## Introduction

**Figure 1:**
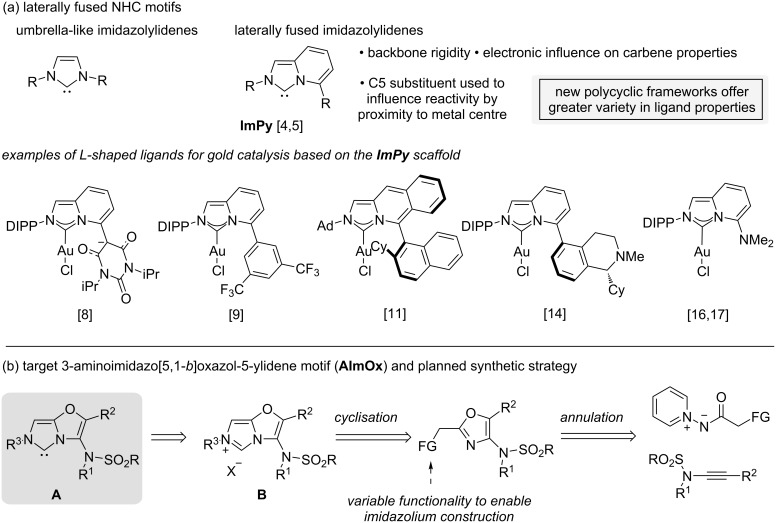
Laterally fused NHC motifs.

Imidazolium-derived nucleophilic heterocyclic carbenes (NHCs) have had a sustained impact across the fields of organometallic and main group chemistry, transition-metal catalysis, materials synthesis and organocatalysis [1]. Laterally annellated polycyclic NHCs offer a useful contrast to the most widely used ‘umbrella-like’ NHCs (Figure 1) [2–3]. An extended π-system influences the donor and acceptor properties of the carbene whilst substitution on the polycycle can position groups adjacent to the active centre.

The imidazo[1,5-*a*]pyridin-3-ylidene motif (**ImPy**), independently introduced by the groups of Lassaletta [4] and Glorius [5], is the most widely explored framework for L-shaped ligands (Figure 1a). Even when only considering gold catalysis [6], the **ImPy** framework has been used to great effect [7]. The motif has been used to introduce sterically demanding NHCs with secondary gold-ligand interactions [8–10], chiral environments [11–13] including those enabling secondary interactions with substrates for asymmetric catalysis [14], cooperative and bimetallic catalysis [7,15], and redox-enabling function for Au(I)/(III) cycles [16–17].

Such L-shaped ligands provide scope to influence the reactivity profile of their resulting metal complexes through steric shielding, direct stabilising interactions with the metal, or by proximal effects to reactive species. Given the sensitivity of metal catalysis to even subtle steric and electronic changes in the ligand sphere, accessing more diverse fused imidazolium frameworks and different peripheral functionality offers significant scope to influence catalytic properties. Few studies into L-shaped imidazolylidines have explored core motifs beyond **ImPy**, with NHCs derived from two π-rich rings fused together particularly underinvestigated [2,18–19].

In this work we report the preparation of a new L-shaped NHC motif, the 3-aminoimidazo[5,1-*b*]oxazol-5-ylidene **A** (shortened hereafter to **AImOx**), which fuses two π-rich rings and positions a sulfonamide group alongside the metal centre (Figure 1b). We envisaged that the potential NHC precursor to **A**, a polysubstituted 3-aminoimidazo[5,1-*b*]oxazol-6-ium motif **B**, might be rapidly accessed from an ynamide by sequential oxazole-forming annulation and imidazolium formation steps. The basis of this approach was a gold-catalysed oxazole formation developed in our group [20–21] that should facilitate access to different groups at the oxazole C-2 position allowing a range of imidazolium-forming cyclisation strategies to be explored. Glorius and co-workers reported the formation of symmetrical NHCs by imidazolium ring formation from bisoxazoline motifs [22] but incorporating the unsaturated oxazole counterparts has not been explored.

## Results and Discussion

**Scheme 1:**
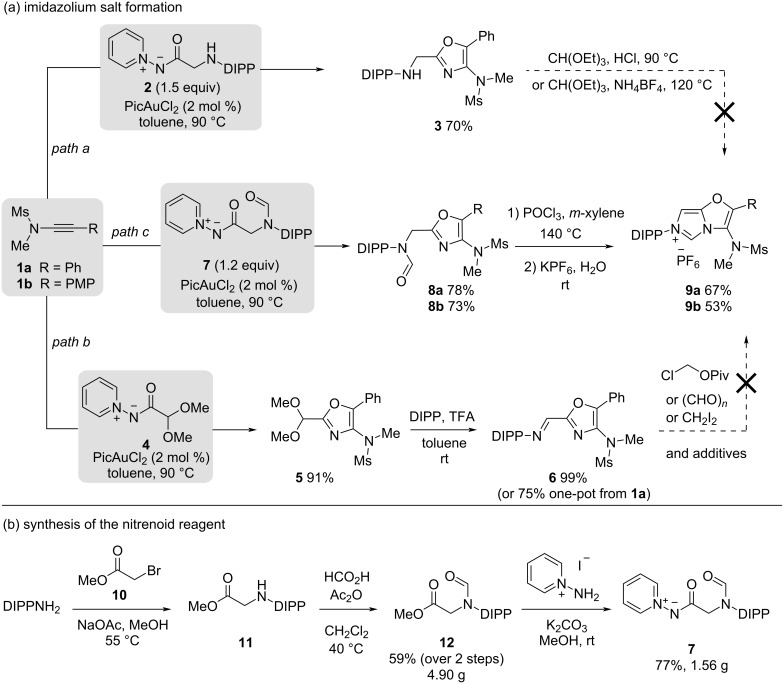
Synthetic studies into the formation of a 3-aminoimdazo[5,1-*b*]oxazol-6-ium motif based on a gold-catalysed oxazole formation. DIPP = 2,6-diisopropylphenylamine; Pic = picolinate; PMP = *p*-methoxyphenyl.

Reaction of ynamide **1a** with the *N*-acylpyridinium-*N*-aminide reagent **2** proceeded in good yield to afford oxazole **3** bearing a C-2 methyleneamino moiety as the first example of a free secondary amine in this annulation type (Scheme 1a, path a). However, attempts to form the desired imidazolium ring from **3** using triethyl orthoformate and different additives were unsuccessful. Similarly, an imine precursor **6**, prepared in high yields by synthesising the known acetal-bearing oxazole **5** [21] and reacting it with 2,6-diisopropylphenylamine, could not be converted into the desired imidazolium salt (Scheme 1a, path b). Applying a range of conditions, including those successful on other annulated systems, led to unreacted starting material or hydrolysis products after work-up (see Supporting Information File 1) [5,18–19,23–27]. The unique Schiff base **6** can however be stored without precautions for several months without degradation and is prepared with minimal processing in 75% yield by telescoping the annulation and condensation steps.

As the 4-aminooxazole motif appeared to be a poor nucleophile, we sought to introduce a formamide motif in place of the amine or imine to allow the use of more forcing cyclisation conditions (Scheme 1a, path c). Oxazole **8a** was obtained in good yield from **1a** using only a slight excess of nitrenoid **7** and 2 mol % catalyst loading. Heating **8a** in the presence of POCl_3_ afforded the 3-aminoimidazo[5,1-*b*]oxazol-6-ium motif, followed by salt metathesis using KPF_6_ leading to the clean hexafluorophosphate salt **9a** in 67% yield after recrystallisation [4].

This two-step assembly of the 3-aminoimidazo[5,1-*b*]oxazol-6-ium motif was also applied to ynamide **1b** affording the PMP-substituted salt **9b** in good yield.

The new nitrenoid reagent **7** is readily prepared from 2,6-diisopropylphenylamine in three steps. Alkylation with methyl bromoacetate is followed by formylation of **11** and then substitution [21] of **12** with *N*-aminopyridinium iodide to yield the bench-stable and crystalline *N*-acylpyridinium aminide **7** in good yield on a gram scale (Scheme 1b).

**Scheme 2:**
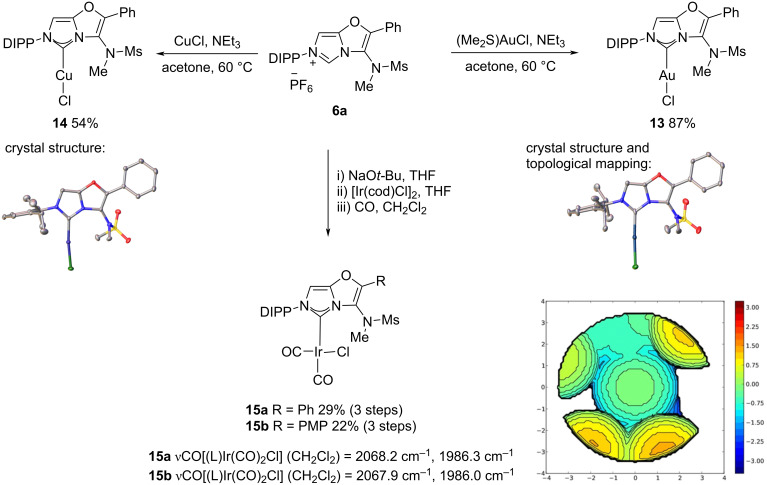
The synthesis of **AImOx**Au(I)Cl, **AImOx**Cu(I)Cl, and **AImOx**Ir(CO)_2_Cl complexes from **6a**. The single crystal X-ray diffraction structures of **13** and **14** have ellipsoids drawn at 50% probability, with hydrogens and solvent omitted for clarity. Selected bond angles and distances: **13**: C1–Au: 1.98 Å, Au–Cl: 2.28 Å, N2–Au: 3.65 Å. N1–C1–N3: 102.7°, N3–C1–Au: 129.5°, N1–C1–Au: 127.1°, C1–Au–Cl: 175.8°. **14**: C1–Au: 1.97 Å, Au–Cl: 2.28 Å, N2–Au: 3.66 Å. N1–C1–N3: 102.6°, N3–C1–Au: 129.4°, N1–C1–Au: 127.9°, C1–Au–Cl: 177.7°. Topographic steric maps of AImOxAuCl **13**. Au–carbene bond selected as *z*-axis, nitrogen’s flanking carbene define *xz* plane. Bondi radii scaled by 1.17, sphere radius 3.5 Å, mesh spacing 0.10, H atoms removed for calculations. Colour coding represents positioning of steric bulk relative to the centre of the sphere, scale in Å.

With the novel 3-aminoimidazo[5,1-*b*]oxazol-6-ium salt in hand, we examined its use as an NHC precursor for the preparation of late transition metal complexes. Treating compound **9a** with triethylamine and either dimethyl sulfide gold(I) chloride or copper(I) chloride in acetone led to the formation of the desired **AImOx**AuCl and **AImOx**CuCl metal chloride complexes **13** and **14**, respectively (Scheme 2) [7].

The ^1^H NMR spectra of the resulting **AImOx** metal complexes show a loss of symmetry for the diisopropyl substituents, indicating restricted rotation about the C(oxazole)–N(sulfonamide) bond. No coalescence is observed at up to 110 °C indicating that these motifs might be useful as a robust atropisomeric system. The molecular structure of **13** and **14** have been unambiguously determined by single crystal X-ray diffraction (Scheme 2) [28]. The N–metal interatomic distances are between 3.53 and 3.66 Å leaving insufficient space for bond rotation about the C–N axis with the sulfonamide substituents being approximately perpendicular to the fused aromatic unit. A percentage buried volume of 44.6% was calculated from the crystal structure of **13** using Cavallo’s method and Sambvca V.2.0 software (Scheme 2) [29]. Although a similar value to that reported for IPrAuCl (%Vbur = 45.4%) [30] the steric map shows a very different steric environment on either side of the ligand.

The **AImOx**Ir(CO)_2_Cl complex **15** was targeted in order to assess the electronic effects of the fused imidazolium core (Scheme 2). No reaction was observed between **6a** and [Ir(cod)Cl]_2_ in the presence of NEt_3_. A solution of the free carbene was prepared from **6** and reacted with [Ir(cod)Cl]_2_ and then CO to afford the **AImOx**Ir(CO)Cl complex **15**. A minor side-product with a strong red colour was formed which could not be fully purified or characterised but has a characteristic AQ quartet of two protons replacing the singlet for the *N*-methyl group in the ^1^H NMR spectra consistent with a cyclometallated complex from C–H insertion [31–32].

Three distinct sets of *N*-methyl and *N*-methylsulfonyl signals, with a major one accounting for approximately 80% of the total, were observed in the ^1^H NMR spectra of **15** likely due to restricted rotation around the metal carbene bond combining with the locked rotation around the oxazole C4–N bond. Elemental analysis was consistent with the proposed structure and only two sharp CO stretching frequencies were observed in the IR (Scheme 2) and so a value for Tolman’s electronic parameter (TEP) could be estimated. [33] At TEP[Ir] = 2053.1 cm^−1^ and 2052.8 cm^−1^ for **15a** and **15b**, respectively, the values for these **AImOx** ligands are towards the electron-deficient end seen with imidazolidines (cf. for IPr TEP[Ir] = 2050.2 cm^−1^) [34].

**Scheme 3:**
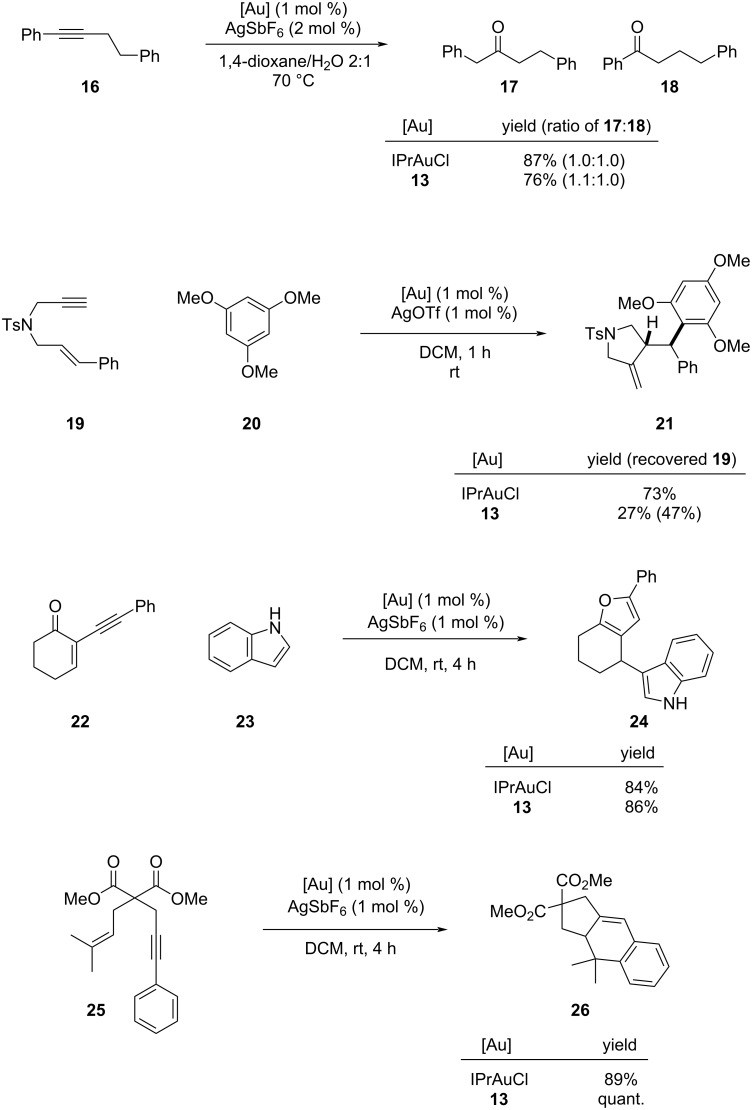
Use of **AImOx**AuCl **13** in catalysis. ^a^Yields are calculated from the ^1^H NMR spectra against an internal standard unless otherwise stated. Isolated yield of **17**:**18** with ratios determined from the ^1^H NMR spectra.

A benchmarking exercise was then performed looking at the reactivity of **13** compared against reaction of symmetrical IPrAuCl across a range of known gold-mediated transformations of alkynes featuring intermolecular attack [35], intramolecular cyclisation [36] or a mixture of both [8,37–39]. The new ligand system proved to deliver competent catalysis. Conversion was seen in all cases at 1 mol % catalyst loading (Scheme 3). Use of **13** resulted in a slight increase of the anti-Markovnikov hydration product **17** over **18** when compared to IPrAuCl [35]. In arylative cyclisations incomplete reaction was seen with enyne **19** [8,37] but ynone **22** [39] afforded high yield of **24**. A quantitative conversion was seen in the intramolecular arylative cyclisation of **25** where **13** outperformed IPrAuCl [36].

## Conclusion

An L-shaped NHC ligand motif, **AImOx**, has been developed and used to access monoligated Au(I), Cu(I) and Ir(I) complexes. The NHC precursors, polysubstituted 3-aminoimidazo[5,1-*b*]oxazol-6-ium salts are readily prepared in an efficient two-step sequence from ynamides using a newly developed nitrenoid reagent **4**. The resulting **AImOx**Au(I) complex is catalytically competent across several transformations with excellent conversions at 1 mol % loading and with broadly comparable reactivity to IPrAuCl. Having validated the **AImOx** motif as a viable ligand platform for development, further elaboration and applications will be reported in due course.

## Supporting Information

File 1 Experimental procedures and characterisation data, additional cyclisation studies, XRD data and NMR spectra of compounds.

## Data Availability

The data generated and analyzed during this study is openly available in the University of Birmingham eData Repository (UBIRA) at https://doi.org/10.25500/edata.bham.00001041.
