# Supplementary material for: A laterally-fused N-heterocyclic carbene framework from polysubstituted aminoimidazo[5,1-b]oxazol-6-ium salts
Source: Beilstein J Org Chem. 2024 Mar 18;20:621–7. doi: 10.3762/bjoc.20.54 (PMC10964033; doi:10.3762/bjoc.20.54)
Supplement: File 1 — Experimental procedures and characterisation data, additional cyclisation studies, XRD data and NMR spectra of compounds. [file Beilstein_J_Org_Chem-20-621-s001.pdf]

BEILSTEIN JOURNAL OF ORGANIC CHEMISTRY

Supporting Information

for

A laterally-fused N-heterocyclic carbene framework from polysubstituted aminoimidazo[5,1-*b*]oxazol-6-ium salts

Andrew D. Gillie, Matthew G. Wakeling, Bethan L. Greene, Louise Male and Paul W. Davies

Beilstein J. Org. Chem. **2024,** *20,* 621–627. doi:10.3762/bjoc.20.54

Experimental procedures and characterisation data, additional cyclisation studies, XRD data and NMR spectra of compounds

License and Terms: This is a supporting information file under the terms of the Creative Commons Attribution License (https://creativecommons.org/licenses/by/4.0). Please note that the reuse, redistribution and reproduction in particular requires that the author(s) and source are credited and that individual graphics may be subject to special legal provisions.
The license is subject to the *Beilstein Journal of Organic Chemistry* terms and conditions: (https://www.beilstein-journals.org/bjoc/terms)

Table of contents

General experimental S1

Starting materials S2

Attempted cyclisation conditions from imine **6** S13

Catalysis S14

NMR spectra S16

Crystallographic data S34

References S37

General experimental

Commercially available chemicals/reagents were purchased from major suppliers (Sigma-Aldrich, Fisher, Acros, Alfa Aesar, Strem, Fluorochem or VWR) and used without further purification. The solvents used were purified using a Pure Solv-MD solvent purification system and were transferred under argon. Anhydrous DMF, 1,4-dioxane and *m*-xylene was dried over 4 Å Linde-type molecular sieves. All reactions were stirred using Teflon-coated magnetic stirrer bars. Asynt DrySyn heating blocks on stirrer hotplates were employed for reactions with temperature controlled via external probe. Reactions were monitored using Merck silica gel 60 F254 TLC plates which were developed using standard visualizing agent: UV fluorescence (254 nm), potassium permanganate /Δ and vanillin /Δ UV. Manual flash column chromatography was carried out on Sigma-Aldrich silica gel (pore size 60 Å, 230–400 mesh particle size); automated flash column chromatography was carried using a Teledyne Isco Combiflash NextGen 100 instrument, using either Teledyne Isco Redisep RediSep® Normal-phase, RediSep Rf Gold® Normal-Phase, or InterChim Puriflash IR Silica flash columns. Melting points were measured in open capillary tubes using a Stuart Scientific melting point apparatus and are uncorrected. Infrared spectra were recorded using a Perkin-Elmer spectrum 100 FTIR spectrometer using an ATR attachment; selected absorbencies (ν_{max}) are reported. Mass spectra were obtained using Waters GCT Premier (EI), Waters LCT (ES) or Waters Synapt (ES). Both the calculated and measured values are reported as neutrals within Waters MassLynx (V4.1 used). High-resolution spectra used a lock-mass to adjust the calibrated mass scale. NMR spectra were recorded using Bruker AV300 or AVIII300 (^{1}H = 300 MHz, ^{19}F = 282 MHz, ^{31}P = 121 MHz) and Bruker AV400 or AVIII400 (^{1}H = 400 MHz, ^{13}C = 101 MHz) spectrometers in commercial solvents at ambient temperature. Chemical shifts (δ) are given in ppm relative to TMS and are calibrated using residual solvent peaks ($CDCl_3$: δ_C = 77.16 ppm, acetone-d_6: δ_C = 206.26 ppm; residual $CHCl_3$ in $CDCl_3$: δ_H = 7.26 ppm, residual acetone in acetone-d_6: δ_H ≡ 2.05 ppm). NMR spectra were run in TMS-free $CDCl_3$, TMS-containing $CDCl_3$ and acetone-d_6. Multiplicity of resonances in ^{1}H NMR spectra were denoted as follows: s (singlet), d (doublet), t (triplet), m (multiplet), *app*. (apparent). Coupling constants (*J*) are quoted to one decimal place. Proton decoupled ^{13}C NMR spectra were recorded using the UDEFT or PENDANT pulse sequences from the Bruker standard pulse program library. ^{13}C DEPT spectra and 2D COSY, HSQC and HMBC spectra were recorded to assist with NMR assignment when necessary. NMR spectra were processed using MestreNova software.

Starting materials

N-Methylmethanesulfonamide (S1)

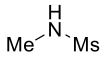

Following a literature procedure[1], $MeNH_2$ (33% in EtOH, 65 mL, 527 mmol) was added to a flask fitted with a reflux condenser and MsCl (9 mL, 116 mmol) was added over 15 minutes whilst cooling with an ice/water bath. The reaction mixture was stirred for 18 h at room temperature before CH_2Cl_2 (150 mL) was added to precipitate $MeNH_3Cl$, which was removed by filtration through a 5 cm pad of silica. The filtrate was concentrated under reduced pressure and purified by flash column chromatography (3:2 EtOAc:hexane) to give *sulfonamide* as a colourless oil (8.34 g, 66%) (*note that this product is water soluble*); IR (neat): ν = 3290, 1405, 1301, 1146, 1126, 1067, 969, 834, 751 cm^{-1}; ^{1}H-NMR (300 MHz, $CDCl_3$): δ = 4.32 (br s, NH), 2.95 (s, 3H), 2.83 (d, *J* = 5.3 Hz, 3H); ^{13}C-NMR (101 MHz, $CDCl_3$): δ = 38.8 (CH_3), 29.5 (CH_3). Spectroscopic data matched those reported[1].

1-(2,2-Dibromovinyl)-4-methoxybenzene (S2)

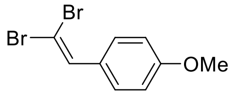

CBr_4 (2.63 g, 7.9 mmol) and PPh_3 (4.23 g, 16.0 mmol) were dissolved in CH_2Cl_2 (0.13 M with respect to aldehyde) under an argon atmosphere whilst cooling with an ice/water bath. The reaction mixture was stirred for 1 hour, after which 4-anisaldehyde (490 µL, 4.0 mmol) was added and stirring was continued for a further 18 h at room temperature. Satd. $NaHCO_{3(aq)}$ solution and additional CH_2Cl_2 were added until all solids were dissolved and the phases were separated. The organic phase was dried over Na_2SO_4, concentrated under reduced pressure. Filtration of the residue through a 5 cm pad of silica (eluting with hexane) afforded the *dibromoalkene* **S2** as a pale yellow solid (1.14 g, 97%); mp: 33-34 °C (lit.[2] 37-38 °C); IR (neat): ν = 2964, 2840, 1603, 1567, 1507, 1254, 1177, 1026, 863 cm^{-1}; ^{1}H-NMR (300 MHz, $CDCl_3$): δ = 7.51 (d, *J* = 8.8 Hz, 2H), 7.41 (s, 1H), 6.89 (d, *J* = 8.8 Hz, 2H), 3.82 (s, 3H); ^{13}C-NMR (101 MHz, $CDCl_3$): δ = 159.8 (C), 136.5 (CH), 130.0 (2CH), 128.0 (C), 113.9 (2CH), 87.4 (C), 55.4 (CH_3). Spectroscopic data matched those reported[3].

N-Methyl-*N*-(phenylethynyl)methanesulfonamide (1a)

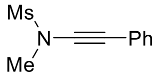

Following a literature procedure [4], a two-necked flask under an argon atmosphere was charged with sulfonamide **S1** (811 mg, 7.4 mmol), K_2CO_3 (2.07 g, 15 mmol), $CuSO_4{\cdot}5H_2O$ (187 mg, 0.75 mmol), 1,10-phenanthroline (270 mg, 1.5 mmol), (bromoethynyl)benzene (1.63 g, 9.0 mmol) and toluene (1 M with respect to amide). The reaction mixture was heated at 70 °C for 18 h. After cooling to room temperature,

the reaction mixture was filtered through a 5 cm pad of silica gel, concentrated under reduced pressure, and then purified by flash column chromatography (10→30% EtOAc in hexane) gave *ynamide* **1a** as a pale yellow solid (1.51 g, 97%); mp: 54-55 °C (lit. [5] 61-63 °C); IR (neat): ν = 2241, 1347, 1320, 1155, 1107, 957, 763 cm^{-1}; ^{1}H-NMR (300 MHz, $CDCl_3$): δ = 7.46-7.37 (m, 2H), 7.36-7.27 (m, 3H), 3.30 (s, 3H), 3.13 (s, 3H); ^{13}C-NMR (101 MHz, $CDCl_3$): δ = 131.7 (2CH), 128.5 (2CH), 128.2 (CH), 122.5 (C), 83.2 (C), 69.6 (C), 39.3 (CH_3), 36.9 (CH_3). Spectroscopic data matched those reported [6].

N-((4-Methoxyphenyl)ethynyl)-*N*-methylmethanesulfonamide (1b)

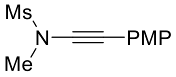

Following a literature procedure [7], a flask under an argon atmosphere was charged with sulfonamide **S1** (218 mg, 2.0 mmol), Cs_2CO_3 (2.60 g, 8.0 mmol), CuI (48 mg, 0.25 mmol), dibromoalkene **S2** (870 mg, 3.0 mmol) and DMEDA (40 µL, 0.37 mmol) and the reaction mixture was heated at 70 °C for 36 h. After cooling to room temperature, the reaction mixture was filtered through a 5 cm pad of silica gel (eluting with CH_2Cl_2), concentrated under reduced pressure and purified by flash column chromatography (20→30% EtOAc in hexane) yielded ynamide **1b** as a white solid (455 mg, 92%); mp: 91-92 °C; IR (neat): ν = 3015, 2935, 2236, 1603, 1512, 1353, 1326, 1246, 1158, 1024, 959, 839 cm^{-1}; ^{1}H-NMR (300 MHz, $CDCl_3$): δ = 7.36 (d, *J* = 8.9 Hz, 2H), 6.83 (d, *J* = 8.9 Hz, 2H), 3.81 (s, 3H), 3.28 (s, 3H), 3.11 (s, 3H); ^{13}C-NMR (101 MHz, $CDCl_3$): δ = 159.8 (C), 133.7 (2CH), 114.4 (C), 114.1 (2CH), 81.8 (C), 69.3 (C), 55.4 (CH_3), 39.4 (CH_3), 36.7 (CH_3). Spectroscopic data matched those reported [5].

Methyl (2,6-diisopropylphenyl)glycinate (11)

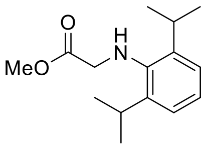

Adapted from a literature procedure[8], methyl bromoacetate (950 µL, 10 mmol) was added to a mixture of 2,6-diisopropylaniline (1.9 mL, 10 mmol) and NaOAc (821 mg, 10 mmol) in methanol (1.5 mL) and the reaction mixture was heated at 55 °C for 6 h. After cooling to room temperature, the reaction mixture was filtered through cotton, concentrated under reduced pressure and purified by flash column chromatography (3:17 Et_2O:hexane) to give *ester* **11** as a red oil (2.20 g, 88%); IR (neat): ν = 2961, 2870, 1742, 1621, 1438, 1344, 1214 cm^{-1}; ^{1}H-NMR (300 MHz, $CDCl_3$): δ = 7.13-7.06 (m, 3H), 3.79 (s, 3H), 3.74 (s, 2H), 3.28 (hept, *J* = 6.8 Hz, 2H), 1.25 (d, *J* = 6.8 Hz, 12H); ^{13}C-NMR (101 MHz, $CDCl_3$): δ = 172.5 (C), 142.6 (C), 142.2 (2C), 124.1 (CH), 123.8 (2CH), 52.4 (CH_2), 52.3 (CH_3), 28.0 (2CH), 24.3 ($4CH_3$); HRMS (EI): *m/z*: calculated for $C_{15}H_{23}NO_2$: 249.1729, found 249.1727 (M).

Methyl *N*-(2,6-diisopropylphenyl)-*N*-formylglycinate (12)

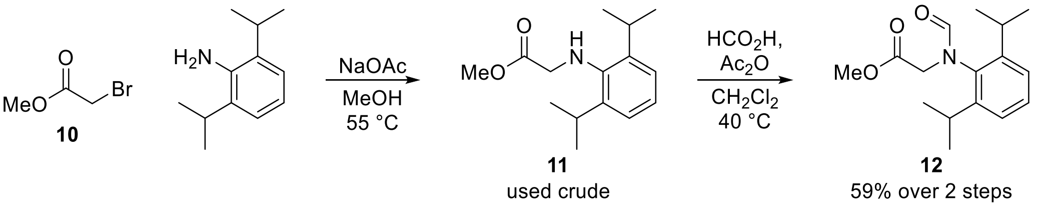

Methyl bromoacetate (2.8 mL, 30.0 mmol) was added to a mixture of 2,6-diisopropylaniline (5.7 mL, 30.0 mmol) and NaOAc (4.92 g, 60.0 mmol) in dry MeOH (4.5 mL) and the reaction mixture was heated at 65 °C for 23 h. After cooling to room temperature, satd. $NaHCO_{3(aq)}$ solution (50 mL) was added and the aqueous phase was extracted with CH_2Cl_2 (3 × 25 mL). The combined organic fractions were washed with brine (50 mL), dried over Na_2SO_4, filtered and concentrated under reduced pressure to give the crude ester **11**. Formic acid (4.5 mL, 119 mmol) and Ac_2O (4.0 mL, 42.4 mmol) were mixed for 1.5 h at room temperature and this mixture was added to a solution of the crude ester **11** in CH_2Cl_2 (15 mL). The resulting reaction mixture was stirred for 21 h at room temperature and 3 h at 40 °C. After cooling to room temperature, satd. $NaHCO_{3(aq)}$ solution (100 mL) was added, the phases were separated and the aqueous phase was extracted with CH_2Cl_2 (3 × 40 mL). The combined organic phases were dried over Na_2SO_4, concentrated under reduced pressure and purified by flash column chromatography (85:15 hexane:EtOAc) to give ester **12** as a white solid (4.90 g, 59% over two steps); mp: 70-72 °C; IR (neat): ν = 2964, 2869, 1763, 1666, 1459, 1329, 1204 cm^{-1}; ^{1}H-NMR (300 MHz, $CDCl_3$): δ = 8.13 (s, 1H), 7.42-7.33 (m, 1H), 7.25-7.17 (m, 2H), 4.21 (s, 2H), 3.76 (s, 3H), 3.23 (hept, *J* = 6.8 Hz, 2H), 1.21 (d, *J* = 6.8 Hz, 6H), 1.17 (d, *J* = 6.8 Hz, 6H); ^{13}C-NMR (101 MHz, $CDCl_3$): δ = 168.2 (C), 163.7 (CH), 148.0 (2C), 135.7 (C), 129.8 (CH), 124.7 (2CH), 52.4 (CH_3), 49.9 (CH_2), 28.2 (2CH), 25.0 (2CH_3), 24.2 (2CH_3); HRMS (ES): *m/z*: calculated for $C_{16}H_{23}NO_3Na$: 300.1576, found 300.1578 (M+Na).

Methyl 2,2-dimethoxyacetate (S3)

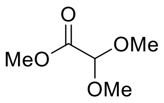

Following a literature procedure [9], $TsOH{\cdot}H_2O$ (400 mg, 2.1 mmol) was added to a solution of glyoxylic acid monohydrate (2.00 g, 22 mmol) in trimethyl orthoformate (12 mL, 109 mmol) and the mixture was stirred for 3 h at room temperature. K_2CO_3 (400 mg, 2.9 mmol) was added and stirring was continued for 0.5 h before isolation of the product by distillation directly from the reaction flask (b.p. 63-64 °C at 25-28 mbar [lit. [9] 64–67 °C at 27 mbar], note that a solvent-containing fraction was collected at 25-28 mbar at room temperature prior to collection of the product fraction) to give ester **S3** as a clear oil

(2.07 g, 71%); IR (neat) ν = 3005, 2955, 2839, 1750, 1440, 1226, 1195, 1116, 1065, 1017, 981, 913, 800 cm^{-1}; ^{1}H-NMR (300 MHz, $CDCl_3$) δ = 4.83 (s, 1H), 3.81 (s, 3H), 3.42 (s, 6H); ^{13}C-NMR (101 MHz, $CDCl_3$) δ = 167.7 (C), 99.0 (CH), 54.1 (2CH_3), 52.6 (CH_3). Spectroscopic data match those reported [9].

General procedure 1 (GP1): synthesis of pyridine-*N*-aminides from esters

N-Aminopyridinium iodide (1.0 equiv) and K_2CO_3 (2.4 equiv) were suspended in MeOH (0.13 M with respect to *N*-aminopyridinium iodide) and the methyl ester (1.2 equiv) was added. The reaction mixture was stirred for 3 days at room temperature at which point the MeOH was removed under reduced pressure and the residue was suspended in 9:1 CH_2Cl_2/MeOH and filtered through a 5 cm pad of basic alumina. The filtrate was concentrated under reduced pressure and, where necessary, purified by flash column chromatography or recrystallisation.

((2,6-Diisopropylphenyl)glycyl)(pyridin-1-ium-1-yl)amide (2)

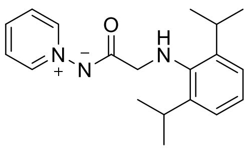

Following **GP1** using *N*-aminopyridinium iodide (1.00 g, 4.5 mmol), K_2CO_3 (1.48 g, 10.7 mmol) and ester **12** (1.35 g, 5.4 mmol). Purification by flash column chromatography (0→10% MeOH in CH_2Cl_2) afforded ylide **2** as red oil (983 mg, 70%); IR (neat): ν = 3360, 2960, 2867, 1577, 1467, 1339, 1269, 1090, 757 cm^{-1}; ^{1}H-NMR (300 MHz, $CDCl_3$): δ = 8.78-8.70 (m, 2H), 8.03-7.90 (m 1H), 7.76-7.64 (m, 2H), 7.15-7.01 (m, 3H), 3.79 (s, 2H), 3.46 (hept, *J* = 6.8 Hz, 2H), 1.28 (d, *J* = 6.8 Hz, 12H); ^{13}C-NMR (101 MHz, $CDCl_3$): δ = 174.0 (C), 144.0 (C), 143.3 (2CH), 142.1 (2C), 137.5 (CH), 126.3 (2CH), 123.7 (2CH), 123.4 (CH), 54.5 (CH_2), 27.9 (2CH), 24.4 (4CH_3); HRMS (ES): *m/z* calculated for $C_{19}H_{25}N_3ONa$: 334.1895, found 334.1909 (M+Na).

N-(2,6-Diisopropylphenyl)-*N*-formylglycyl)(pyridin-1-ium-1-yl)amide (7)

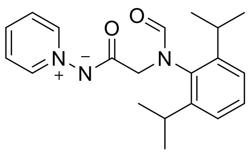

Following **GP1** using *N*-aminopyridinium iodide (1.33 g, 6.00 mmol), K_2CO_3 (1.82 g, 6.60 mmol) and ester **11** (1.83 g, 6.60 mmol). Purification by flash column chromatography (94:6 CH_2Cl_2/MeOH) afforded ylide **7** as brown solid (1.56 g, 77%); mp: 170-172 °C; IR (neat): ν = 2963, 2927, 2869, 1670, 1590, 1469, 1349, 1259, 1192 cm^{-1}; *NMR shows a mixture of two rotamers in a ~ 0.3:0.7 ratio:* ^{1}H-NMR (300 MHz, $CDCl_3$): δ = 8.71-8.62 (m, 2H), 8.68 and 8.17 (s, 1H), 7.93 and 7.87 (tt, *J* = 7.7, 1.2 Hz, 1H), 7.70-7.57 (m, 2H), 7.37-7.26 (m, 1H), 7.23-7.15 (m, 2H), 4.25 and 4.13 (s, 2H), 3.47 and 3.17 (hept, *J* = 6.8 Hz, 2H), 1.28-1.08 (m, 12H); ^{13}C-NMR (101 MHz, $CDCl_3$): δ = 171.6 and 171.2 (C), 164.7 and 163.8 (CH), 148.2and 146.6 (2C),

143.0 and 142.7 (2CH), 137.8 and 137.1 (CH), 136.9 and 136.1 (C), 129.3 and 128.8 (CH), 126.4 and 126.2 (2CH), 124.4 and 124.2 (2CH), 54.8 and 52.8 (CH_2), 28.6 and 28.0 (2CH), 25.1 and 24.6 ($2CH_3$), 24.4 and 24.2 ($2CH_3$); HRMS (ES): *m/z* calculated for $C_{20}H_{25}N_3O_2Na$: 362.1844, found 362.1832 (M+Na).

(2,2-Dimethoxyacetyl)(pyridin-1-ium-1-yl)amide (4)

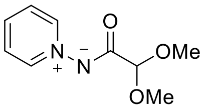

Following **GP1** using *N*-aminopyridinium iodide (666 mg, 3.0 mmol), K_2CO_3 (995 mg, 7.2 mmol) and ester **S3** (486 mg, 3.6 mmol). Purification by flash column chromatography (100:0:1→90:10:1 CH_2Cl_2:MeOH:NEt_3) yielded ylide **4** as a white powder (496 mg, 84%); mp: 90-92 °C; IR (neat): ν = 3110, 3086, 2968, 2926, 2832, 1617, 1600, 1568, 1467, 1399, 1339, 1268, 1198, 1151, 1106, 1044, 983, 900, 851, 779, 749 cm^{-1}; ^1H-NMR (300 MHz, $CDCl_3$): δ = 8.62-8.57 (m, 2H), 7.86 (tt, *J* = 7.7, 1.1 Hz, 1H), 7.64-7.55 (m, 2H), 4.76 (s, 1H), 3.39 (s, 6H); ^{13}C-NMR (101 MHz, $CDCl_3$): δ = 170.7 (C), 142.9 (2CH), 137.6 (CH), 126.1 (2CH), 102.0 (CH), 53.7 ($2CH_3$); HRMS (ES): *m/z* calculated. for $C_9H_{13}N_2O_3$: 197.0926, found 197.0929 (M+H).

General procedure 2 (GP2): gold-catalysed oxazole synthesis

Pyridinium ylide (1.2–1.5 equiv), dichloro(2-pyridinecarboxylato)gold (1–5 mol %), and ynamide (1.0 equiv) were added to a flask under an argon atmosphere. Toluene (0.1 M with respect to ynamide) was added (where ynamides were oils these were added as a solution in toluene) and the reaction mixture was heated at 90 °C for the indicated time. After cooling to room temperature, the reaction mixture was filtered through a 5 cm plug of silica gel (washing with CH_2Cl_2 and then EtOAc) and the filtrate was concentrated under reduced pressure and purified by flash column chromatography.

N-(2-(((2,6-Diisopropylphenyl)amino)methyl)-5-phenyloxazol-4-yl)-*N*-methyl-methanesulfonamide (3)

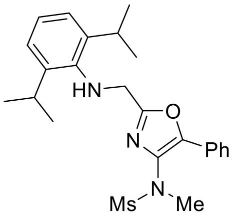

Following **GP2** using ylide **2** (402 mg, 1.3 mmol), ynamide **1a** (180 mg, 0.86 mmol) and gold catalyst (6.7 mg, 2 mol%) for 17 h. Purification by flash column chromatography (15→20% EtOAc in hexane) yielded oxazole **3** as a white solid (266 mg, 70%); mp: 160-162 °C; IR (neat): ν = 2966, 1452, 1364, 1342, 1069, 969 cm^{-1}; ^1H-NMR (300 MHz, $CDCl_3$): δ = 7.93-7.83 (m, 2H), 7.50-7.33 (m, 3H), 7.17-7.07 (m, 3H), 4.24 (s, 2H), 3.36 (hept, *J* = 6.8 Hz, 2H), 3.27 (s, 3H), 3.11 (s, 3H), 1.27 (d, *J* = 6.8 Hz, 12H); ^{13}C-NMR (101 MHz, $CDCl_3$): δ = 159.7 (C), 146.0 (C),

142.8 (2C), 141.6 (C), 133.2 (C), 129.2 (CH), 128.9 (2CH), 126.7 (C), 125.4 (2CH), 124.8 (CH), 123.9 (2CH), 48.2 (CH_2), 38.0 (CH_3), 36.9 (CH_3), 27.9 (2CH), 24.3 ($4CH_3$); HRMS (ES): *m/z* calculated for $C_{24}H_{31}N_3O_3SNa$: 464.1984, found 464.1995 (M+Na).

N-(2-(Dimethoxymethyl)-5-phenyloxazol-4-yl)-N-methylmethanesulfonamide (5)

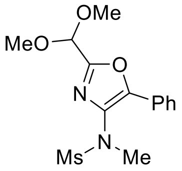

Note: To avoid degradation, room temperature water baths should be used when evaporating solvent from this compound.

Following **GP2** with ylide **4** (295 mg, 1.5 mmol), ynamide **1a** (209 mg, 1.0 mmol) and gold catalyst (8.2 mg, 2 mol%) for 2.5 h. Purification by flash column chromatography (80:20:1→50:50:1 hexane:EtOAc:NEt_3) yielded oxazole **5** as a white solid (296 mg, 91%); mp: 108-110 °C; IR (neat): ν = 3016, 2956, 2909, 2833, 1451, 1358, 1337, 1324, 1152, 1116, 1059, 961, 841, 763 cm^{-1}; ^{1}H-NMR (300 MHz, $CDCl_3$): δ = 7.98-7.93 (m, 2H), 7.48-7.41 (m, 2H), 7.41-7.34 (m, 1H), 5.50 (s, 1H), 3.49 (s, 6H), 3.29 (s, 3H), 3.14 (s, 3H); ^{13}C-NMR (101 MHz, $CDCl_3$): δ = 156.4 (C), 146.5 (C), 133.2 (C), 129.5 (CH), 128.9 (2CH), 126.5 (C), 125.7 (2CH), 97.0 (CH), 53.9 ($2CH_3$), 38.0 (CH_3), 37.2 (CH_3); HRMS (ES): *m/z* calculated for $C_{14}H_{18}N_2O_5SNa$: 349.0834, found 349.0840 (M+Na).

(E)-N-(2-(((2,6-Diisopropylphenyl)imino)methyl)-5-phenyloxazol-4-yl)-N-methyl-methanesulfonamide (6)

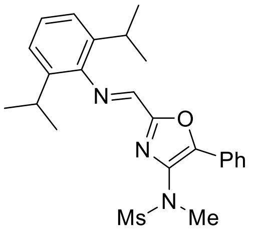

Trifluoroacetic acid (3.75 mL, 49 mmol) was added to a solution of oxazole **5** (248 mg, 0.76 mmol) and diisopropylaniline (160 µL, 0.85 mmol) in toluene (7.5 mL). The reaction mixture was stirred for 14 h at room temperature before cooling in an ice/water bath whilst NEt_3 (7.5 mL, 54 mmol) was added over 10 minutes resulting in a colour change from red to yellow. The solvent was removed under reduced pressure and the crude product was purified by flash column chromatography (80:20:1 hexane:EtOAc:NEt_3) to give oxazole **6** as a yellow solid (331 mg, 99%); mp: 198-202 °C; IR (neat): ν = 2967, 1618, 1451, 1345, 1154, 970 cm^{-1}; ^{1}H-NMR (400 MHz, $CDCl_3$): δ = 8.14-8.08 (m, 2H), 8.02 (s, 1H), 7.53-7.40 (m, 3H), 7.22-7.14 (m, 3H), 3.37 (s, 3H), 3.21 (s, 3H), 2.97 (hept, J = 6.9 Hz, 2H), 1.21 (d, J = 6.9 Hz, 12H); ^{13}C-NMR (101 MHz, $CDCl_3$): δ = 155.0 (C), 148.6 (CH), 148.2 (C), 147.8 (C), 137.4 (2C), 135.4 (C), 130.3 (CH), 129.1 (2CH), 126.3 (2CH), 126.1 (C), 125.4 (CH), 123.4 (2CH), 38.1 (CH_3), 37.2 (CH_3), 28.1 (2CH), 23.8 ($4CH_3$); HRMS (ES): *m/z* calculated for $C_{24}H_{30}N_3O_3S$: 440.2008, found 440.1997 (M+H).

N-(2,6-Diisopropylphenyl)-*N*-((4-(*N*-methylmethylsulfonamido)-5-phenyloxazol-2-yl)methyl)formamide (8a)

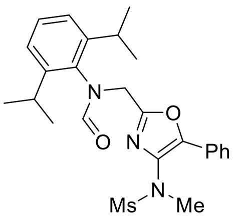

Following **GP2** using ylide **7** (611 mg, 1.8 mmol), ynamide **1a** (314 mg, 1.5 mmol) and gold catalyst (12 mg, 2 mol%) for 4 h. Purification by flash column chromatography (25→50% EtOAc in hexane) yielded oxazole **8a** as a white solid (549 mg, 78%); mp: 193-195 °C; IR (neat): ν = 2971, 2855, 1675, 1495, 1348, 1153 cm^{-1}; NMR *shows a mixture of two rotamers in a ~ 0.2:0.8 ratio:* ^{1}H-NMR (300 MHz, $CDCl_3$): δ = 8.75 and 8.19 (s, 1H), 7.88-7.80 (m, 2H), 7.48-7.31 (m, 4H), 7.20 (d, *J* = 7.7 Hz, 2H), 4.90 and 4.75 (s, 2H), 3.20 and 3.19 (s, 3H), 3.06 and 2.98 (s, 3H), 2.96 and 2.81 (hept, *J* = 6.9 Hz, 2H), 1.18-1.03 (m, 12H); ^{13}C-NMR (101 MHz, $CDCl_3$): *only the peaks for the major rotamer are reported* δ = 163.5 (CH), 155.9 (C), 148.1 (2C), 146.8 (C), 134.3 (C), 133.4 (C), 130.1 (CH), 129.4 (CH), 129.0 (2CH), 126.5 (C), 125.5 (2CH), 124.7 (2CH), 43.9 (CH_2), 38.0 (CH_3), 36.9 (CH_3), 28.5 (2CH), 25.3 ($2CH_3$), 23.8 ($2CH_3$); HRMS (ES): *m/z* calculated for $C_{25}H_{31}N_3O_4SNa$: 492.1933, found 492.1943 (M+Na).

N-(2,6-Diisopropylphenyl)-*N*-((5-(4-methoxyphenyl)-4-(*N*-methylmethylsulfon-amido)oxazol-2-yl)methyl)formamide (8b)

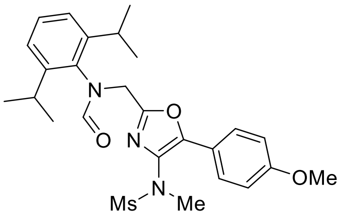

Following **GP2** using ylide **7** (1.84 g, 5.4 mmol), ynamide **1b** (1.08 g, 4.5 mmol) and gold catalyst (35 mg, 2 mol%) for 4 h. Purification by flash column chromatography (30→50% EtOAc in hexane) yielded oxazole **8b** as a white solid (1.64 g, 73%); mp: 148-151 °C; IR (neat): ν = 2969, 1686, 1509, 1345, 1177, 1151, 837 cm^{-1}; *NMR shows a mixture of two rotamers in a ~ 0.3:0.7 ratio:* ^{1}H-NMR (300 MHz, $CDCl_3$): δ = 8.75 and 8.18 (s, 1H), 7.82-7.75 (m, 2H), 7.41-7.31 (m, 1H), 7.20 (d, *J* = 7.7 Hz, 2H), 6.99-6.90 (m, 2H), 4.89 and 4.73 (s, 2H), 3.85 and 3.84 (s, 3H), 3.18 and 3.16 (s, 3H), 3.04 and 2.96 (s, 3H), 2.95 and 2.81 (hept, *J* = 6.9 Hz, 2H), 1.19-1.02 (m, 12H); ^{13}C-NMR (101 MHz, $CDCl_3$): *only the peaks for the major rotamer are reported* δ = 163.3 (CH), 160.4 (C), 155.0 (C), 148.0 (2C), 146.9 (C), 134.2 (C), 131.8 (C), 130.0 (CH), 127.1 (2CH), 124.6 (2CH), 119.2 (C), 114.3 (2CH), 55.4 (CH_3), 43.7 (CH_2), 37.9 (CH_3), 36.7 (CH_3), 28.4 (2CH), 25.3 ($2CH_3$), 23.6 ($2CH_3$); HRMS (ES): *m/z* calculated for $C_{26}H_{34}N_3O_5S$: 500.2219, found 500.2215 (M+H).

General procedure 3 (GP3): Synthesis of AImOx·HPF$_6$ salts

Adapted from a literature procedure[10], under an argon atmosphere $POCl_3$ (3.0 equiv) was added to a solution of oxazole (1.0 equiv) in *m*-xylene (0.1 M with respect to oxazole) and the reaction mixture was heated at 140 °C for 64 h. After cooling to room temperature, the impure **AImOx**·HCl salt was isolated by flash column chromatography (0→10% MeOH in CH_2Cl_2). The resulting brown solid was taken up in refluxing H_2O (≈ 10 mL) and the hot solution was filtered through glass wool, the flask was then washed out with additional boiling H_2O (≈ 5 mL) which was also filtered through the glass wool. The aqueous filtrate was allowed to cool to room temperature and a saturated solution of $KPF_{6(aq)}$ (2.0 equiv) was added, leading to instant precipitation of the crude AImOx·HPF_6 salt. The solid was collected by filtration, washed with ice-cold water and Et_2O, and then recrystallised from acetone by addition of Et_2O to give the **AImOx**·HPF_6 salt.

6-(2,6-Diisopropylphenyl)-3-(*N*-methylmethylsulfonamido)-2-phenylimidazo[5,1-*b*]oxazol-6-ium hexafluorophosphate(V) (9a)

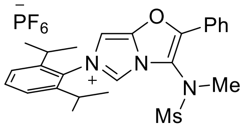

Following **GP3** using oxazole **8a** (827 mg, 1.8 mmol) and $POCl_3$ (500 µL, 5.4 mmol), purification by flash column chromatography and ion exchange with KPF_6 (644 mg, 3.5 mmol) gave **9a** as a white solid (706 mg, 67%); mp: 236-238 °C; IR (neat): ν = 3148, 2967, 1622, 1346, 1236, 1156, 1061, 830 cm^{-1}; ^{1}H-NMR (400 MHz, d_6-acetone): δ = 9.77 (d, J = 1.6 Hz, 1H), 8.11-8.08 (m, 2H), 8.06 (d, J = 1.6 Hz, 1H), 7.72-7.65 (m, 4H), 7.51 (d, J = 7.9 Hz, 2H), 3.58 (s, 3H), 3.37 (s, 3H), 2.63 (hept, J = 6.8 Hz, 2H), 1.22 (d, J = 6.8 Hz, 6H), 1.20 (d, J = 6.8 Hz, 6H); ^{13}C-NMR (101 MHz, d_6-acetone): δ = 152.4 (C), 146.9 (2C), 145.7 (C), 133.08 (CH), 133.05 (CH), 132.6 (C), 130.6 (2CH), 127.7 (2CH), 125.7 (C), 125.6 (2CH), 121.8 (CH), 119.2 (C), 101.4 (CH), 40.1 (CH_3), 37.1 (CH_3), 29.2 (2CH), 24.7 (2CH_3), 24.3 (2CH_3); ^{19}F{^{1}H}-NMR (282 MHz, d_6-acetone): δ = -72.6 (d, $J_{F\text{-}P}$ = 708 Hz); ^{31}P{^{1}H}-NMR (121 MHz, d_6-acetone): δ = -145.2 (hept, $J_{F\text{-}P}$ = 708 Hz); HRMS (ES): *m/z* calculated for $C_{25}H_{30}N_3O_3S$: 452.2008, found 452.2004 (M-PF_6); Anal. calculated for $C_{25}H_{30}F_6N_3O_3PS$: C, 50.25; H, 5.06; N, 7.03. Found: C, 50.19; H, 4.86; N, 7.10.

6-(2,6-Diisopropylphenyl)-2-(4-methoxyphenyl)-3-(*N*-methylmethylsulfon-amido)imidazo[5,1-*b*]oxazol-6-ium hexafluorophosphate(V) (9b)

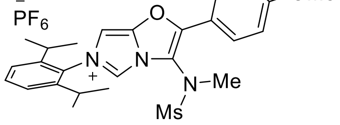

Following **GP3** using oxazole **8b** (343 mg, 0.7 mmol) and $POCl_3$ (200 µL, 2.1 mmol), purification by flash column chromatography and ion exchange with KPF_6 (254 mg,

1.4 mmol) gave **9b** as a white solid (232 mg, 53%); mp: 146-148 °C; IR (neat): ν = 3148, 2969, 1620, 1608, 1346, 1261, 1178, 1155, 830 cm^{-1}; ^{1}H-NMR (400 MHz, $CDCl_3$): δ = 8.51 (d, *J* = 1.5 Hz, 1H), 7.84 (d, *J* = 9.0 Hz, 2H), 7.56 (t, *J* = 7.9 Hz, 1H), 7.33 (d, *J* = 7.9 Hz, 2H), 7.09 (d, *J* = 9.0 Hz, 2H), 7.03 (d, *J* = 1.5 Hz, 1H), 3.89 (s, 3H), 3.42 (s, 3H), 3.18 (s, 3H), 2.39 (hept, *J* = 6.8 Hz, 2H), 1.21 (d, *J* = 6.8 Hz, 6H), 1.18 (d, *J* = 6.8 Hz, 6H); ^{13}C-NMR (101 MHz, $CDCl_3$): δ = 162.9 (C), 152.5 (C), 146.0 (2C), 143.8 (C), 132.4 (CH), 131.0 (C), 128.9 (2CH), 124.8 (2CH), 119.9 (CH), 116.4 (C), 116.1 (C), 115.4 (2CH), 99.4 (CH), 55.7 (CH_3), 40.2 (CH_3), 36.9 (CH_3), 28.7 (2CH), 24.6 ($2CH_3$), 24.2 ($2CH_3$); ^{19}F{^{1}H}-NMR (282 MHz, $CDCl_3$): δ = -72.9 (d, $J_{F\text{-}P}$ = 713 Hz); ^{31}P{^{1}H}-NMR (121 MHz, $CDCl_3$): δ = -144.6 (hept, $J_{F\text{-}P}$ = 713 Hz); HRMS (ES): *m/z* calculated for $C_{26}H_{32}N_3O_4S$: 482.2114, found 482.2117 (M-PF_6); Anal. calculated for $C_{26}H_{32}F_6N_3O_4PS$: C, 49.76; H, 5.14; N, 6.70. Found: C, 49.81; H, 5.15; N, 6.77.

General procedure 4 (GP4): Synthesis of (AImOx)MCl complexes

Adapted from a literature procedure [11], a vial was charged sequentially with AImOx·HPF_6, the corresponding metal chloride, NEt_3 (70 µL, 0.50 mmol) and acetone (2 mL) and heated at 60 °C for 18 h. The solvent was removed under reduced pressure and the crude mixture was taken up in CH_2Cl_2 (≈ 10 mL) and washed with H_2O (2 × 10 mL). The organic phase was dried over Na_2SO_4, concentrated under reduced pressure and purified by flash column chromatography to give the (AImOx)MCl complex.

AImOxAuCl (13)

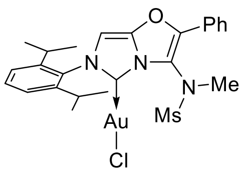

Following **GP4** using **9a** (100 mg, 0.17 mmol) and (DMS)AuCl (59.2 mg, 0.20 mmol). Purification by flash column chromatography (30→50% EtOAc in hexane) gave **13** as a white solid (99.6 mg, 87%); mp: decomp. 230 °C; IR (neat): ν = 3144, 2968, 1621, 1351, 1158, 1055, 965 cm^{-1}; ^{1}H-NMR (400 MHz, $CDCl_3$): δ = 8.01-7.95 (m, 2H), 7.59-7.49 (m, 4H), 7.28 (d, *J* = 7.8 Hz, 2H), 6.79 (s, 1H), 3.73 (s, 3H), 3.53 (s, 3H), 2.48-2.31 (m, 2H), 1.34-1.28 (m, 6H), 1.20-1.14 (m, 6H); ^{13}C-NMR (101 MHz, $CDCl_3$): δ = 154.6 (C), 151.0 (C), 145.9 (C), 145.7 (C), 144.5 (C), 135.1 (C), 131.5 (CH), 131.2 (CH), 129.4 (2CH), 126.1 (2CH), 125.4 (C), 124.5 (2CH), 118.0 (C), 97.4 (CH), 43.0 (CH_3), 37.8 (CH_3), 28.6 (2CH), 24.7 ($2CH_3$), 24.6 (CH_3), 24.5 (CH_3); HRMS (ES): *m/z* calculated for $C_{25}H_{29}Au^{35}ClN_3O_3SNa$: 706.1181, found 706.1204 (M+Na); Anal. calculated for $C_{25}H_{29}AuClN_3O_3S$: C, 43.90; H, 4.27; N, 6.14. Found: C, 44.10;

H, 4.17; N, 6.15. Crystals suitable for single crystal X-ray diffraction were grown by slow evaporation of a solution of **13** in EtOAc.

AImOxCuCl (14)

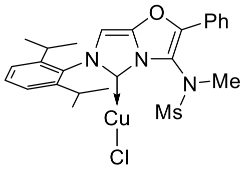

Following **GP4** using **9a** (100 mg, 0.17 mmol) and CuCl (34 mg, 0.34 mmol). Purification by flash column chromatography (30% EtOAc in hexane) gave **14** as a white solid (49.9 mg, 54%); mp: 216-217 °C; IR (neat): ν = 3149, 2962, 2926, 2869, 1620, 1351, 1157, 1057, 1036, 964 cm^{-1}; ^{1}H-NMR (400 MHz, $CDCl_3$): δ = 8.01-7.95 (m, 2H), 7.58-7.48 (m, 4H), 7.29 (d, *J* = 8.0 Hz, 2H), 6.76 (s, 1H), 3.73 (s, 3H), 3.48 (s, 3H), 2.44 (hept, *J* = 6.8 Hz, 1H), 2.35 (hept, *J* = 6.8 Hz, 1H), 1.29 (d, *J* = 6.8 Hz, 3H), 1.26 (d, *J* = 6.8 Hz, 3H), 1.19 (d, *J* = 6.8 Hz, 3H), 1.17 (d, *J* = 6.8 Hz, 3H); ^{13}C-NMR (101 MHz, $CDCl_3$): δ = 159.2 (C), 150.5 (C), 145.9 (C), 145.7 (C), 145.1 (C), 135.5 (C), 131.3 (CH), 131.1 (CH), 129.4 (2CH), 126.1 (2CH), 125.6 (C), 124.4 (2CH), 118.1 (C), 97.5 (CH), 43.1 (CH_3), 37.3 (CH_3), 28.6 (2CH), 25.1 (CH_3), 25.0 (CH_3), 24.5 (CH_3), 24.2 (CH_3); Anal. calculated for $C_{25}H_{29}ClCuN_3O_3S$: C, 54.54; H, 5.31; N, 7.63. Found: C, 54,76; H, 5.32; N, 7.70. Crystals suitable for single crystal X-ray diffraction were grown by diffusion of pentane into a solution of **14** in $CH_2Cl_2/CDCl_3$.

(9a)Ir(CO)$_2$Cl (15a)

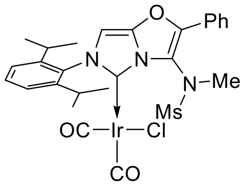

Inside a N_2 filled glovebox NaO*t*-Bu (21.0 mg, 0.22 mmol) was added to a solution of **9a** (119 mg, 0.20 mmol) in THF (2 mL) and the suspension was stirred at room temperature for 20 minutes before filtration through a 3 cm pad of celite (washing with 1 mL of THF) into a vial containing $[Ir(cod)Cl]_2$ (71.8 mg, 0.107 mmol, 1.07 equiv) and the resulting suspension was stirred for 1 hour at room temperature. The vial was removed from the glovebox and the contents filtered through a 3 cm pad of silica gel and the filtrate purified by flash column chromatography (4:1 hexane:EtOAc). The resulting yellow solid (**9a)Ir(cod)Cl** was dissolved in CH_2Cl_2 (3 mL) and CO (balloon pressure) was bubbled through the stirred solution for 5 minutes. The solvent was removed under reduced pressure and the resulting solid purified by flash column chromatography (3:1 hexane:EtOAc) to give **15a** as a yellow solid (43.0 mg, 29% over two steps); mp: 110-112 °C; IR (CH_2Cl_2): ν = 2068.19 (CO), 1986.32 (CO) cm^{-1}; *Restricted rotation around the metal–carbene bond results in 3 sets of signals (1 major, 2 minor)*; ^{1}H-NMR (400 MHz, $CDCl_3$): δ = 8.00-7.92 (m, 2H), 7.61-7.50 (m, 4H), 7.37-7.28 (m, 2H), 6.80 (s, 1H), 3.75_{minor} and 3.68_{major} and 3.63_{minor} (s, 3H), 3.71_{minor} and 3.47_{major} and 3.43_{minor} (s, 3H),

2.87-2.76 (m, 1H), 2.53-2.41 (m, 1H), 1.44-1.40$_{minor}$ and 1.36-1.27$_{major}$ (m, 6H), 1.13 (d, *J* = 6.7 Hz, 3H), 1.11-1.04 (m, 3H); ^{13}C-NMR (101 MHz, $CDCl_3$) *Only the major rotamer is reported*: δ = 180.4 (CO), 167.7 (CO), 159.8 (C), 150.3 (C), 147.2 (C), 145.6 (C), 145.3 (C), 136.1 (C), 131.3 (CH), 131.0 (CH), 129.5 (2CH), 126.7 (2CH), 125.5 (C), 124.5 (CH), 123.8 (CH), 119.7 (C), 99.0 (CH), 42.4 (CH_3), 38.1 (CH_3), 28.6 (2CH), 27.3 (CH_3), 26.4 (CH_3), 23.5 (CH_3), 22.1 (CH_3); Anal. calculated for $C_{27}H_{29}ClIrN_3O_5S$: C, 44.10; H, 3.98; N, 5.71. Found: C, 44.29; H, 4.06; N, 5.43.

(9b)Ir(CO)$_2$Cl (15b)

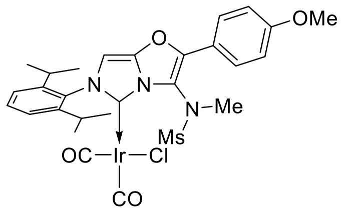

Inside a N_2 filled glovebox NaO*t*-Bu (21.0 mg, 0.22 mmol) was added to a solution of **9b** (125 mg, 0.20 mmol) in THF (2 mL) and the suspension was stirred at room temperature for 20 minutes before filtration through a 3 cm pad of celite (washing with 1 mL of THF) into a vial containing $[Ir(cod)Cl]_2$ (70.0 mg, 0.104 mmol, 1.04 equiv) and the resulting suspension was stirred for 1 hour at room temperature. The vial was removed from the glovebox and the contents filtered through a 3 cm pad of silica gel and the filtrate purified by flash column chromatography (4:1 hexane:EtOAc). The resulting yellow solid (**9b)Ir(cod)Cl** was dissolved in CH_2Cl_2 (3 mL) and CO (balloon pressure) was bubbled through the stirred solution for 5 minutes. The solvent was removed under reduced pressure and the resulting solid purified by flash column chromatography (3:1 hexane:EtOAc) to give **15b** as a yellow solid (34.2 mg, 22% over two steps); mp: 114-116 °C; IR (CH_2Cl_2): ν = 2067.92 (CO), 1985.97 (CO) cm^{-1}; *Restricted rotation around the metal-carbene bond results in 3 sets of signals (1 major, 2 minor)*; ^{1}H-NMR (400 MHz, $CDCl_3$): δ = 7.94-7.87 (m, 2H), 7.56-7.49 (m, 1H), 7.36-7.28 (m, 2H), 7.10-7.03 (m, 2H), 6.78 (s, 1H), 3.89 (s, 3H), 3.74$_{minor}$ and 3.68$_{major}$* and 3.62$_{minor}$ (s, 3H), 3.68$_{minor}$* and 3.46$_{major}$ and 3.42$_{minor}$ (s, 3H), 2.88-2.74 (m, 1H), 2.55-2.41 (m, 1H), 1.44-1.39 and 1.35-1.27 (m, 6H), 1.16-1.10 (m, 3H), 1.10-1.03 (m, 3H); ^{13}C-NMR (101 MHz, $CDCl_3$) *Only the major rotamer is reported*: δ = 180.4 (CO), 167.7 (CO), 161.9 (C), 159.3 (C), 150.5 (C), 147.3 (C), 145.5 (C), 145.4 (C), 136.1 (C), 131.0 (CH), 128.4 (2CH), 124.5 (CH), 124.4 (C), 123.8 (CH), 117.8 (C), 114.9 (2CH), 98.9 (CH), 55.6 (CH_3), 42.4 (CH_3), 38.0 (CH_3), 28.62 (CH), 28.61 (CH), 27.3 (CH_3), 26.4 (CH_3), 23.5 (CH_3), 22.1 (CH_3); Anal. calculated for $C_{28}H_{31}ClIrN_3O_6S$: C, 43.94; H, 4.08; N, 5.49. Found: C, 44.25; H, 4.22; N, 5.32. *The overlap of these two signals was determined using an HSQC spectrum.

Attempted cyclisation conditions from imine 6

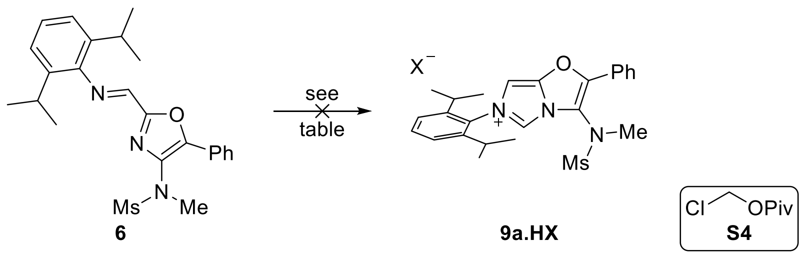

Entry	Reagent	Solvent	Temperature[a]	X	Yield
1	**S4**	MeCN	81 °C	Cl	0%
2	**S4**	Ph_2O	195 °C	Cl	0%
3	**S4**, AgOTf	CH_2Cl_2	40 °C	OTf	0%
4	**S4**, AgOTf	1,4-dioxane	120 °C	OTf	0%
5	**S4**, NaI	MeCN	70 °C	I	0%
6	**S4**, NaI, $ZnCl_2$	MeCN	70 °C	I	0%
7	$(CH_2O)_n$, $ZnCl_2$, HCl	THF	60 °C	Cl	0%
8	$(CH_2O)_n$, TMSCl	EtOAc	77 °C	Cl	0%
9	CH_2I_2	MeCN	100 °C	I	0%
10	CH_2I_2, 2AgOTf	toluene	110 °C	I	0%

[a] Reactions above the normal boiling point of the solvent were carried out in a sealed tube

Catalysis

Catalysis starting material synthesis

Catalysis starting materials were prepared according to the literature: **16** [12], **19** [13], **22** [14] and **24** [15].

Hydration of alkyne 16

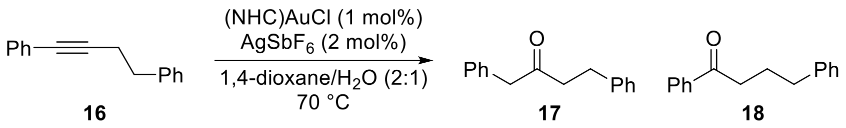

Following a literature procedure [16], $AgSbF_6$ (6.9 mg, 2 mol %), (NHC)AuCl (1 mol %), degassed 1,4-dioxane (670 µL), alkyne **16** (206 mg, 1.0 mmol) and degassed H_2O (330 µL) were added to a Schlenk tube under an argon atmosphere and the reaction mixture was heated at 70 °C for 17 h. H_2O (5 mL) was added and the mixture was extracted with CH_2Cl_2 (2 × 5 mL). The combined organic extracts were dried over Na_2SO_4 and purified by flash column chromatography (19:1 hexane:EtOAc) to give a mixture of ketones **17** and **18** as a clear oil. Product ratios were determined by ^{1}H-NMR spectroscopy with comparison to the literature spectra for **17** [17] and **18** [18].

Using IPrAuCl gave 196.7 mg (87%) with a 1.0:1.0 ratio of **17** and **18**; using **13** gave 167.9 mg (76%) with a 1.1:1.0 ratio of **17** and **18**.

Arylative cyclisations

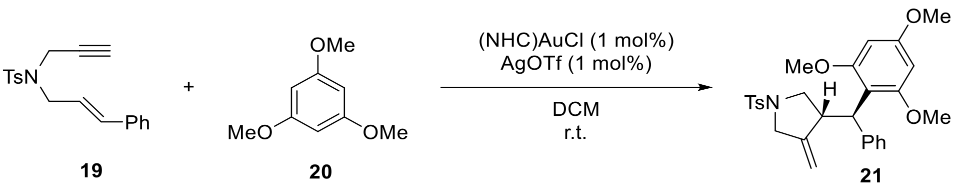

Following an adapted literature procedure [19], AgOTf (0.51 mg, 1 mol %), (NHC)AuCl (4 mM in CH_2Cl_2, 0.5 mL, 2 µmol) and CH_2Cl_2 (0.5 mL) were added to a vial and stirred at room temperature for 10 minutes. The catalytic mixture was then added to a vial containing **19** (65.1 mg, 0.2 mmol), **20** (100.9 mg, 0.6 mmol) in CH_2Cl_2 (1 mL). The reaction was stirred at room temperature for 1 hour. The mixture was filtered through a short pad of silica (eluting with 1:1 hexane/EtOAc) and the solvents evaporated under reduced pressure. Product yield was determined by ^{1}H-NMR spectroscopy relative to an internal standard 1,2,4,5-tetramethylbenzene and with comparison to the literature spectra for **21** [19].

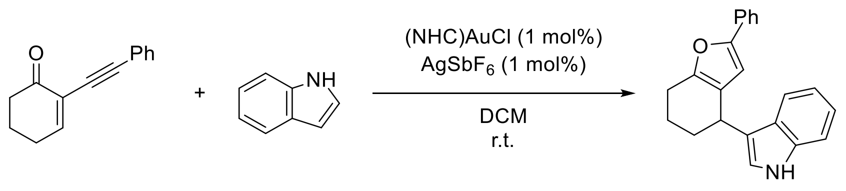

22 **23** **24**

Following an adapted literature procedure [15], enone **22** (19.6 mg, 0.1 mmol), indole **23** (11.7 mg, 0.1 mmol), (NHC)AuCl (2 mM in CH_2Cl_2, 0.5 mL, 1 µmol) and $AgSbF_6$ (2 mM in CH_2Cl_2, 0.5 mL, 1 µmol)) were added to a vial. The reaction was stirred for 4 h at rt. The reaction was diluted with Et_2O (2 mL) and passed through a pad of silica eluting with Et_2O (3 × 1 mL). The solvent was evaporated under reduced pressure. Product yield was determined by ^{1}H-NMR spectroscopy relative to an internal standard 1,2,4,5-tetramethylbenzene and with comparison to the literature spectra for **24** [20].

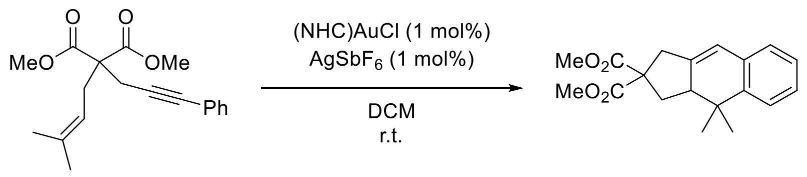

25 **26**

Following an adapted literature procedure [21], enyne **25** (31.4 mg, 0.1 mmol), (NHC)AuCl (2 mM in CH_2Cl_2, 0.5 mL, 1 µmol) and $AgSbF_6$ (2 mM in CH_2Cl_2, 0.5 mL, 1 µmol)) were added to a vial. The reaction was stirred for 4 h at rt. The reaction was diluted with Et_2O (2 mL) and passed through a pad of silica eluting with Et_2O (3 × 1 mL). The solvent was evaporated under reduced pressure. Product yield was determined by ^{1}H-NMR spectroscopy relative to an internal standard 1,2,4,5-tetramethylbenzene and with comparison to the literature spectra for **26** [21].

NMR spectra

((2,6-Diisopropylphenyl)glycyl)(pyridin-1-ium-1-yl)amide (2) in $CDCl_3$ ^1H and ^{13}C PENDANT NMR

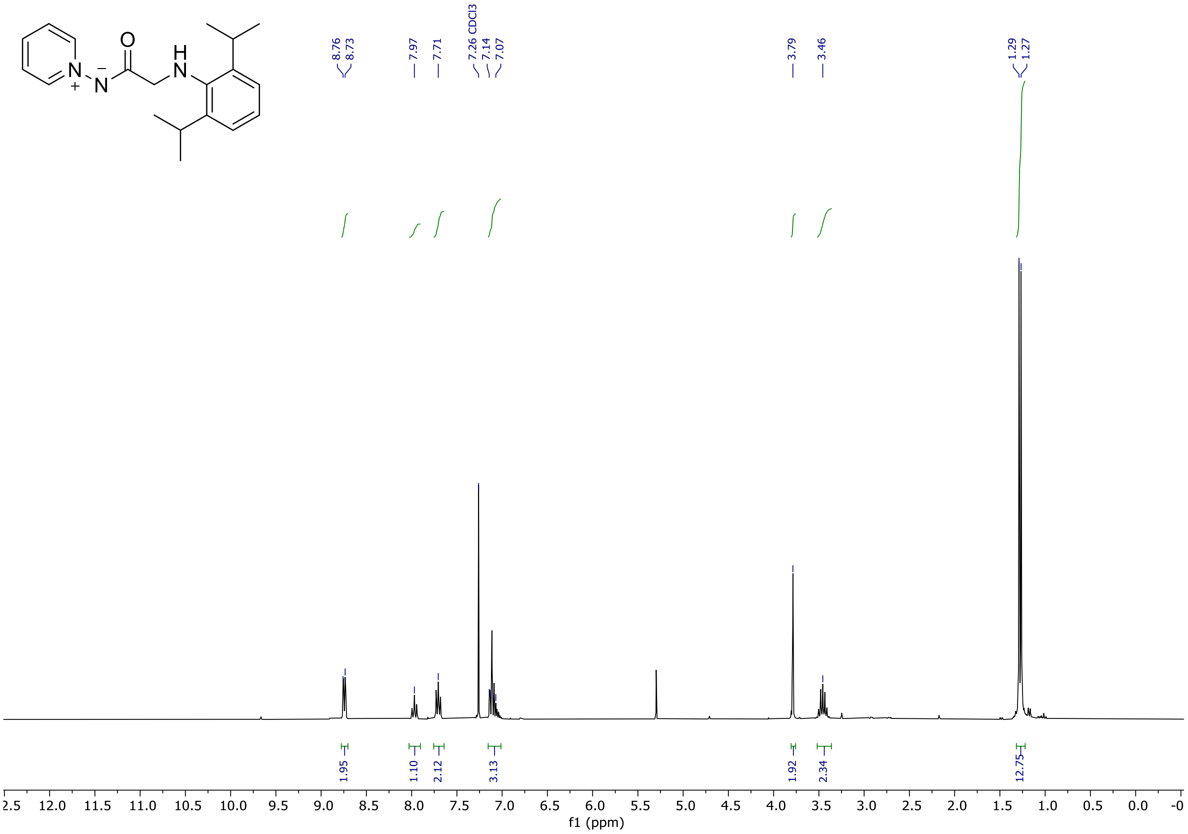

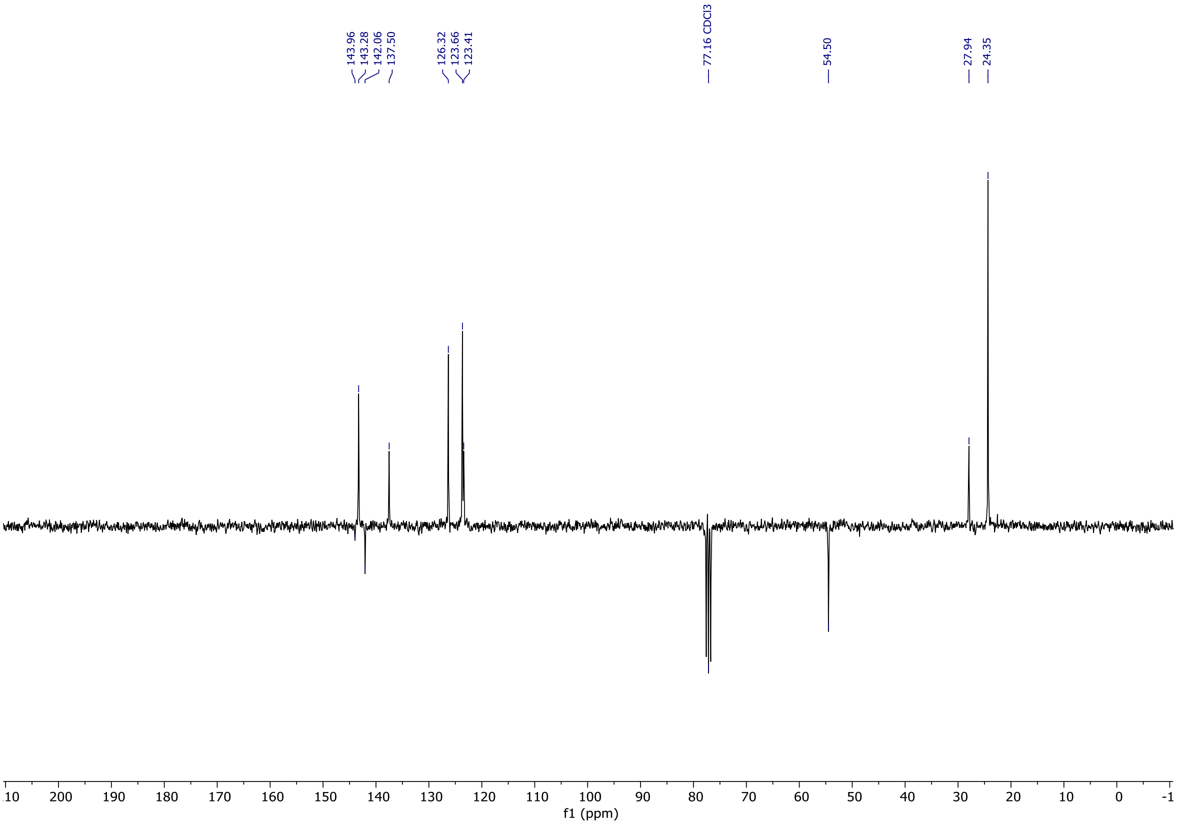

N-(2-(((2,6-Diisopropylphenyl)amino)methyl)-5-phenyloxazol-4-yl)-N-methylmethanesulfonamide (3) in $CDCl_3$ ^1H and ^{13}C PENDANT NMR

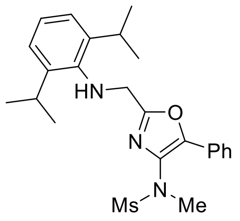

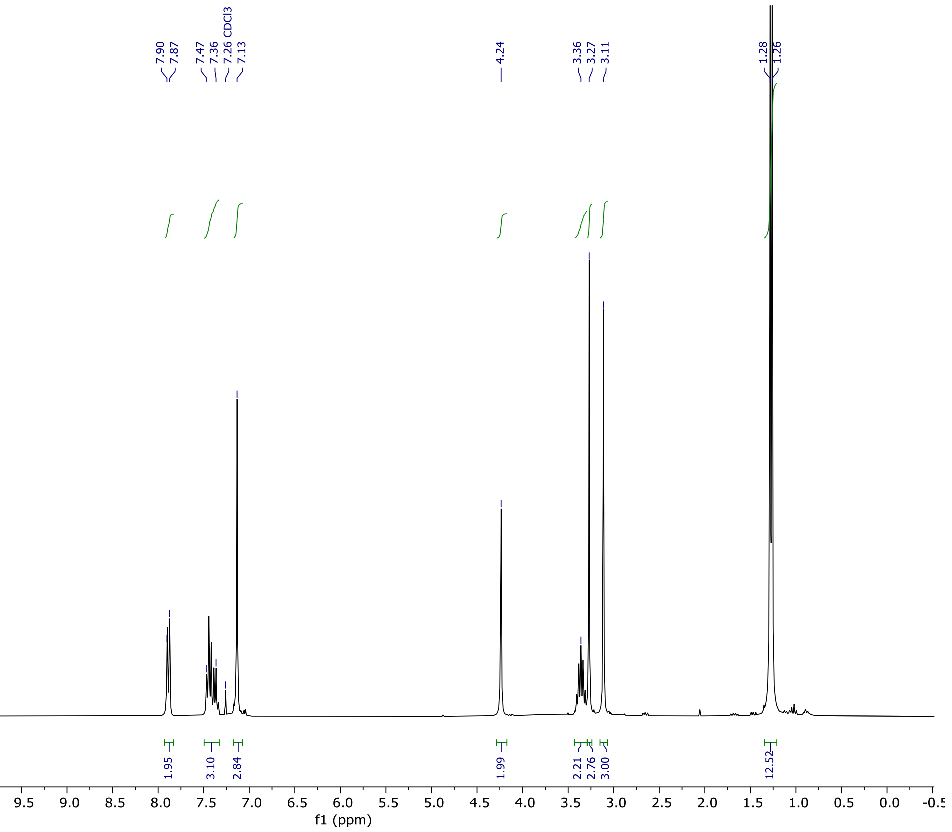

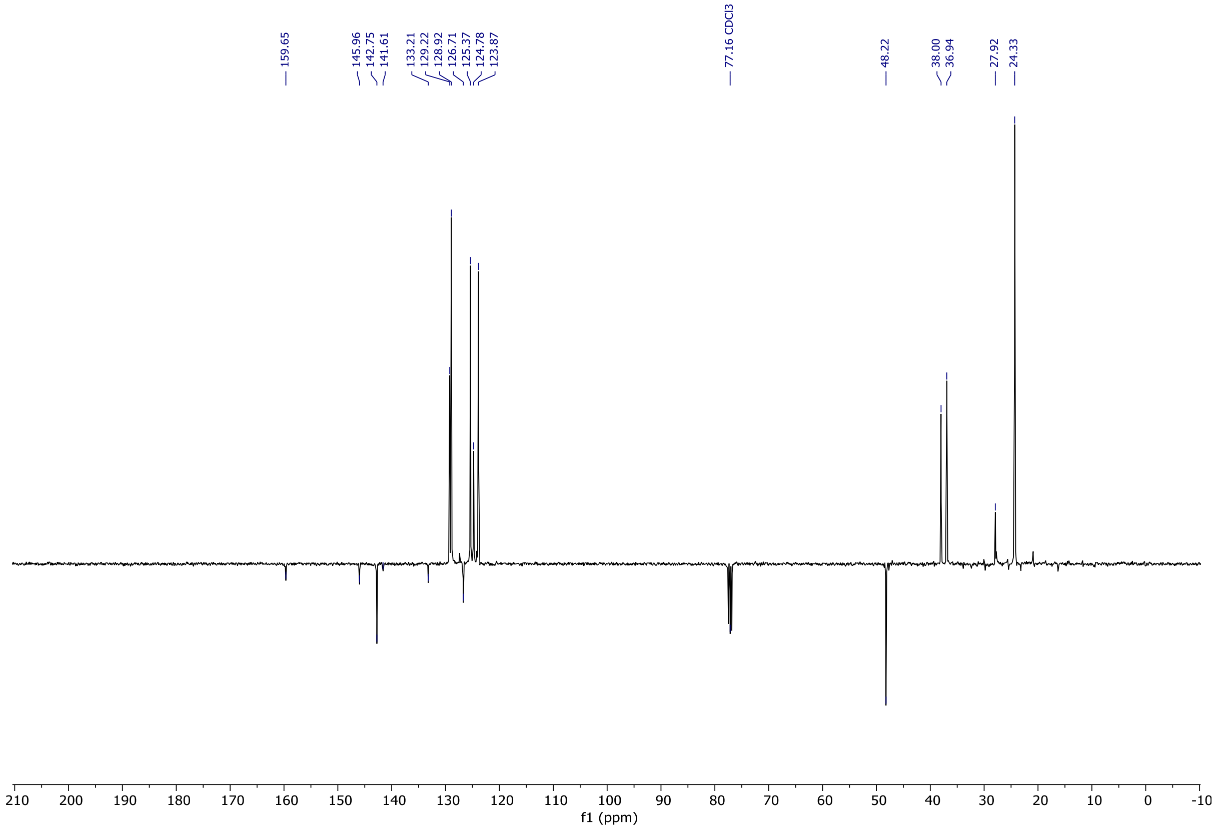

N-(2,6-Diisopropylphenyl)-N-formylglycyl)(pyridin-1-ium-1-yl)amide (7) in $CDCl_3$ ^1H and ^{13}C PENDANT NMR

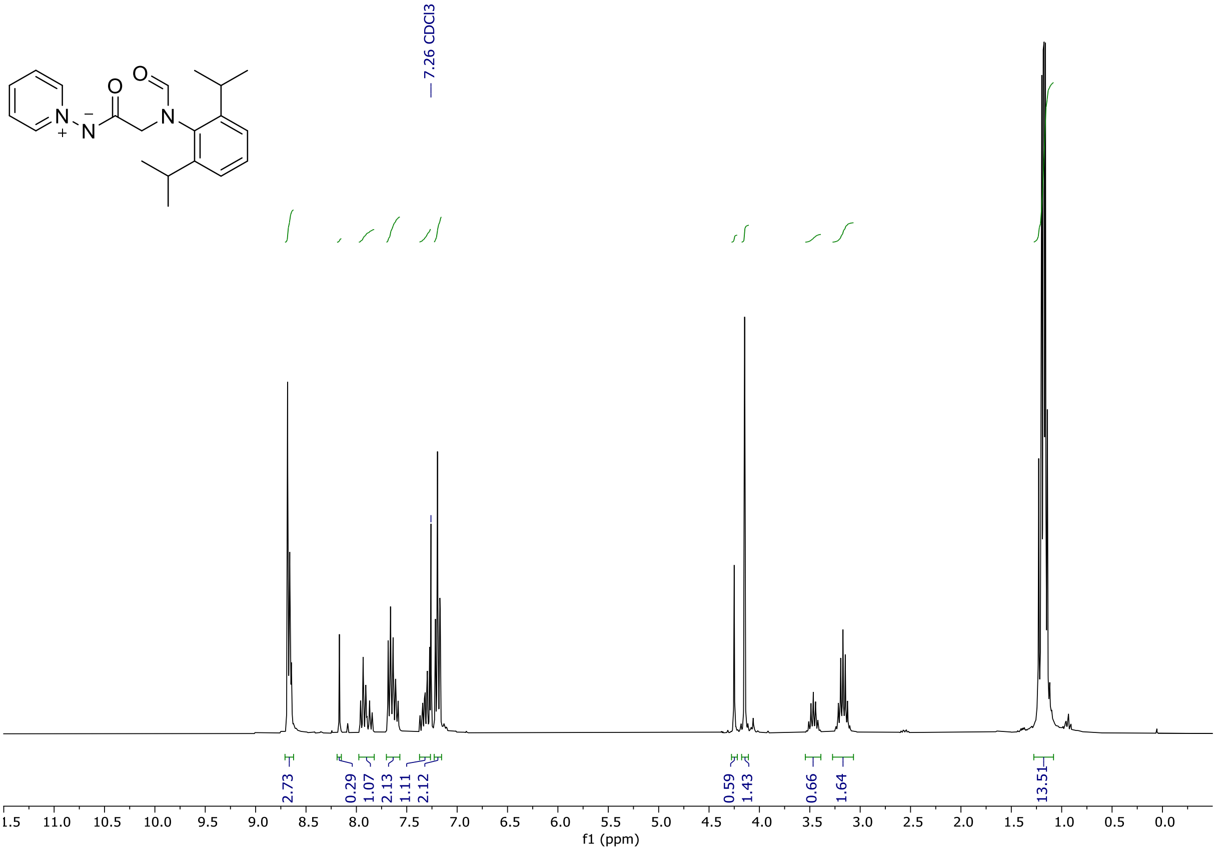

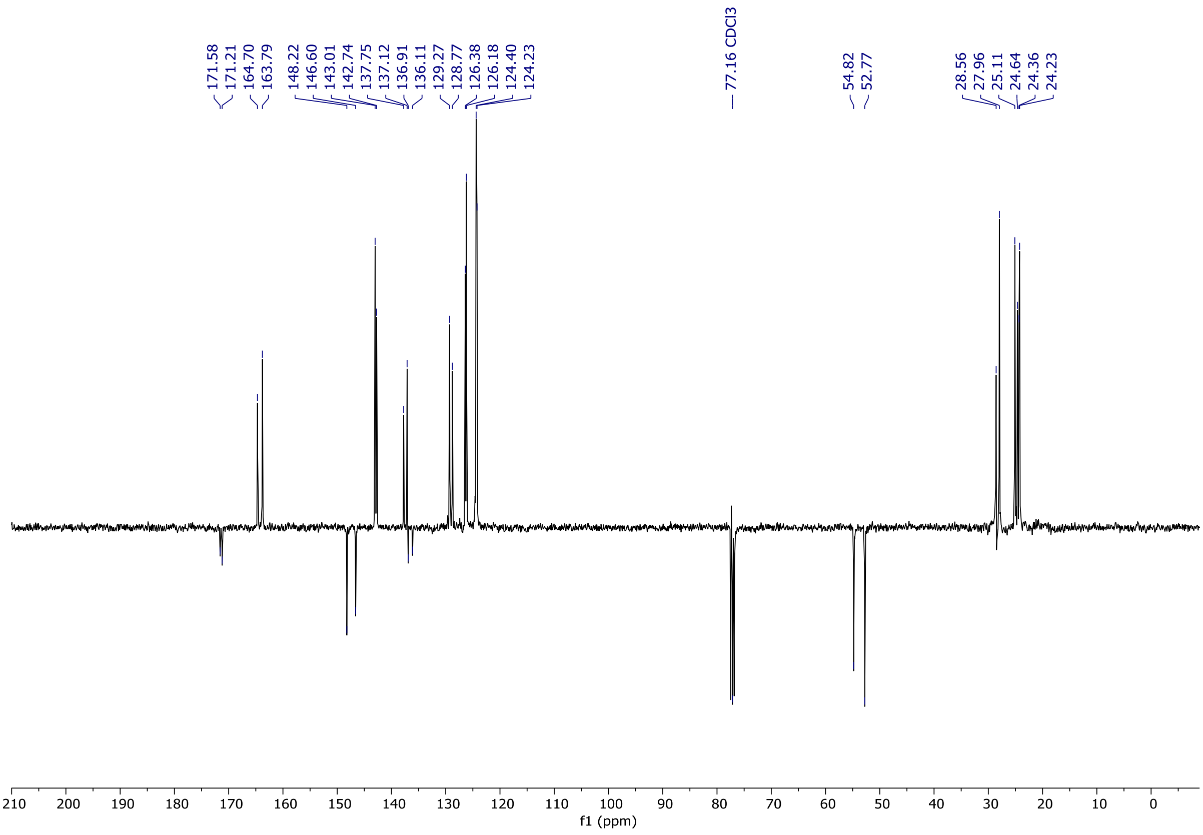

N-(2,6-Diisopropylphenyl)-N-((4-(N-methylmethylsulfonamido)-5-phenyloxazol-2-yl)methyl)formamide (8a) in $CDCl_3$ ^1H and ^{13}C PENDANT NMR

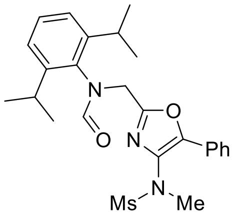

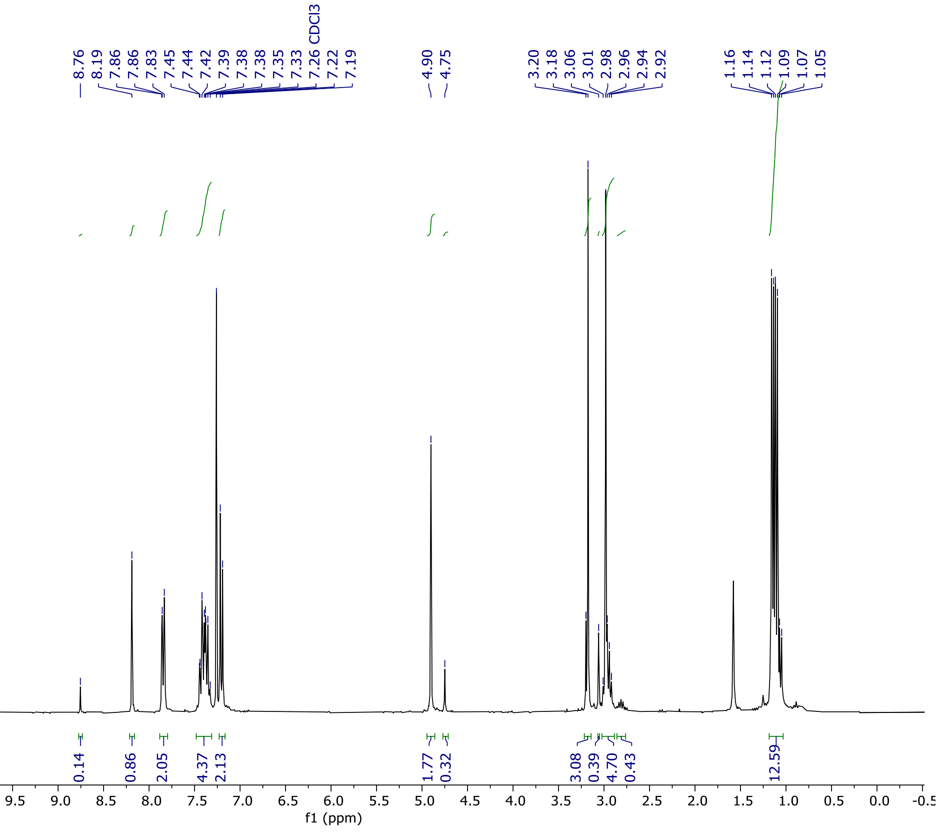

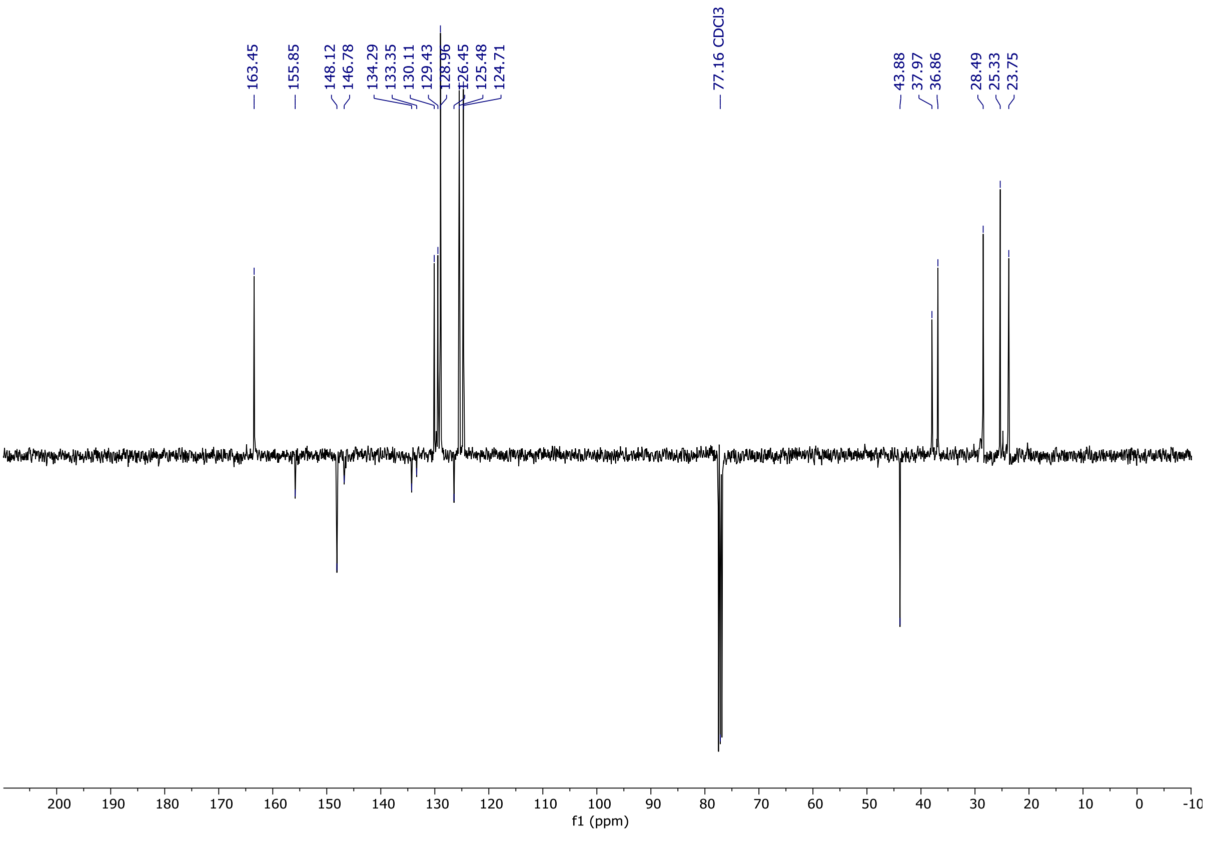

N-(2,6-Diisopropylphenyl)-N-((5-(4-methoxyphenyl)-4-(N-methylmethylsulfon-amido)oxazol-2-yl)methyl)formamide (8b) in $CDCl_3$ ^{1}H and ^{13}C PENDANT NMR

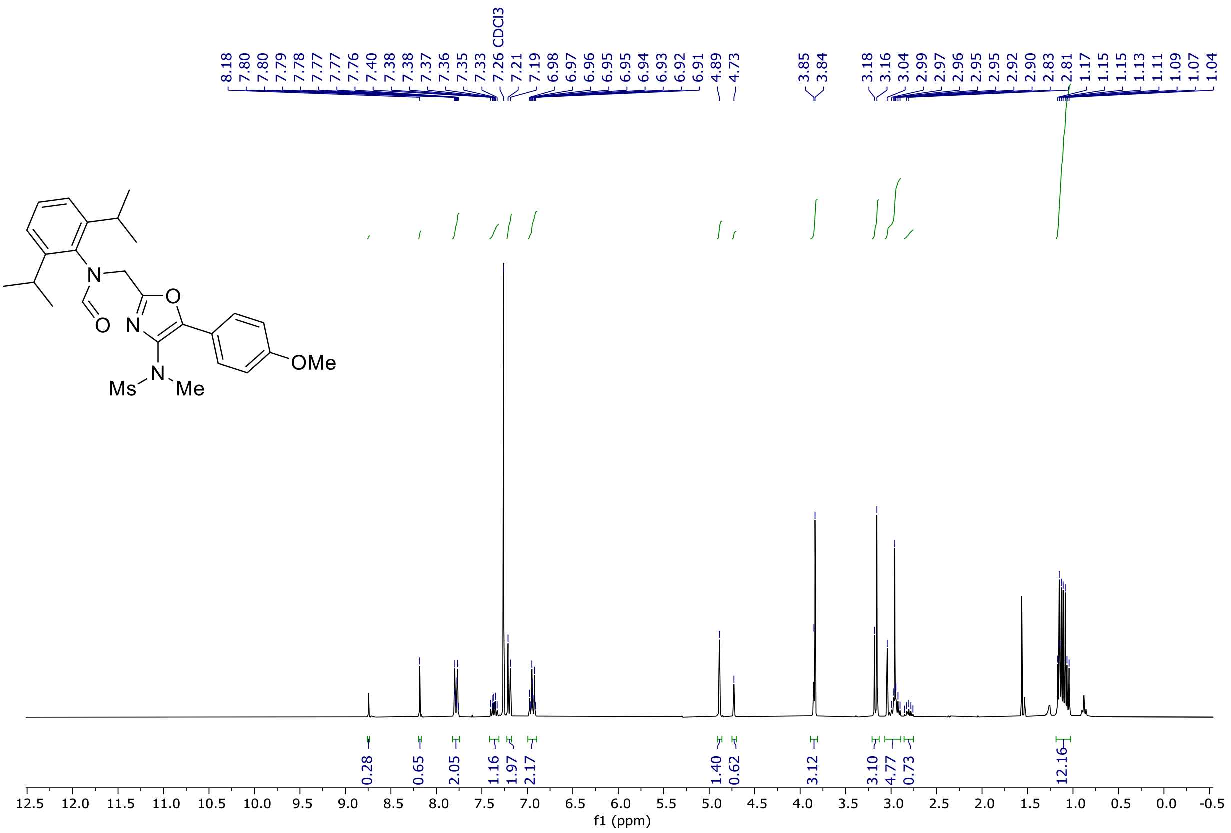

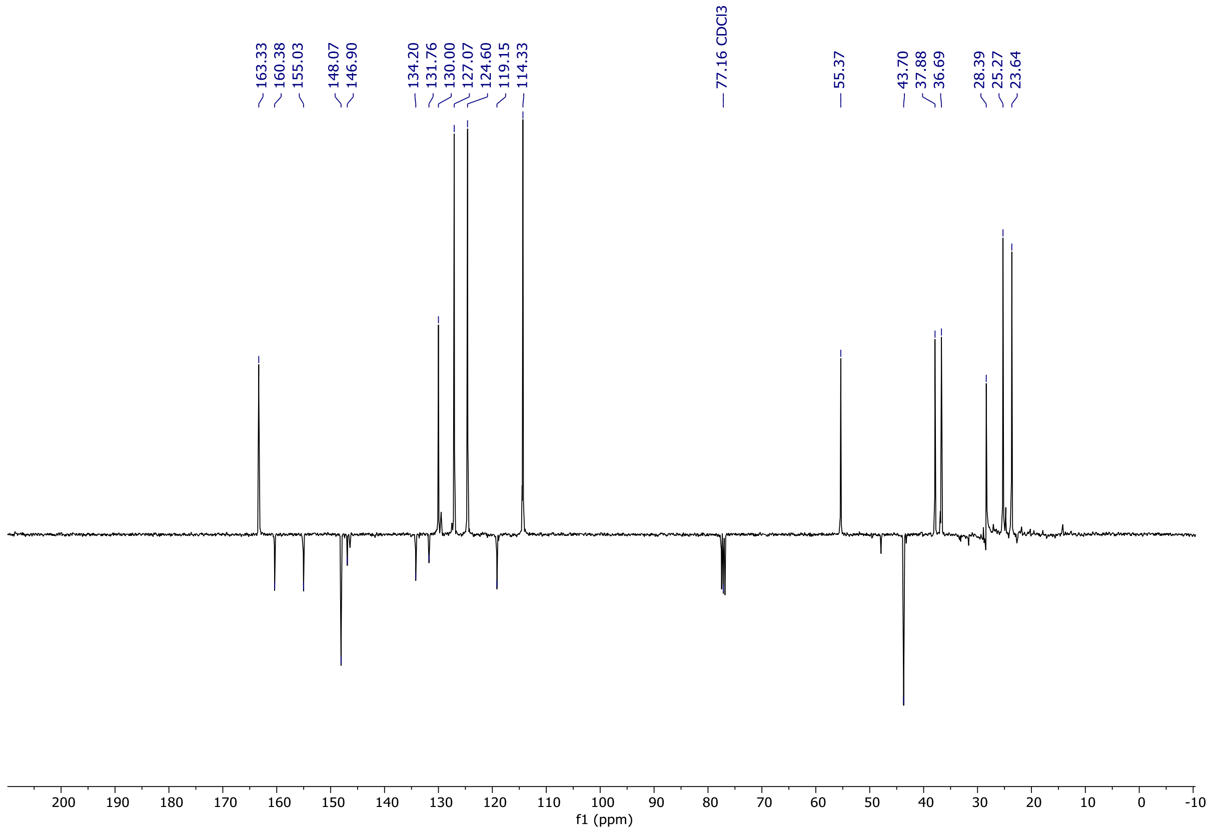

6-(2,6-Diisopropylphenyl)-3-(*N*-methylmethylsulfonamido)-2-phenylimidazo[5,1-*b*]oxazol-6-ium hexafluorophosphate(V) (9a) in d^6-acetone ^{1}H and ^{13}C UDEFT NMR

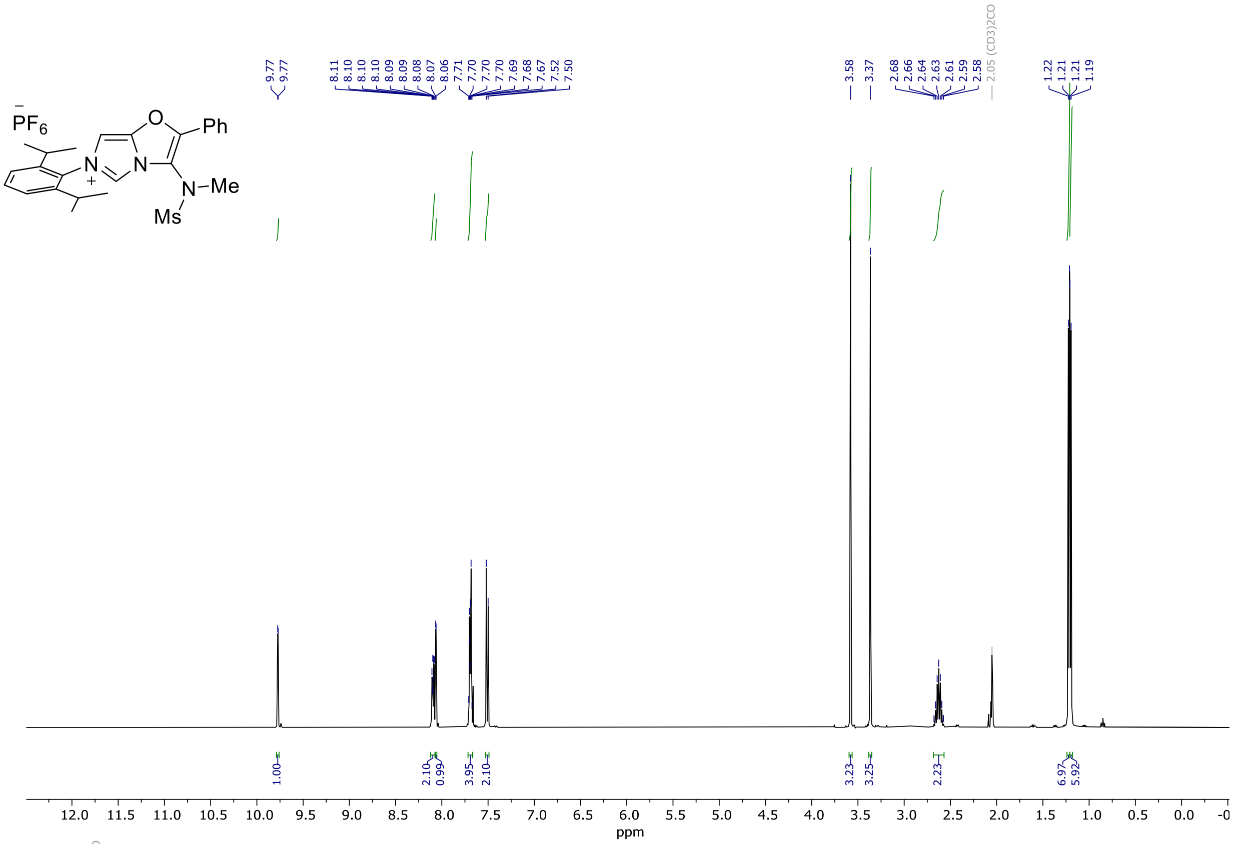

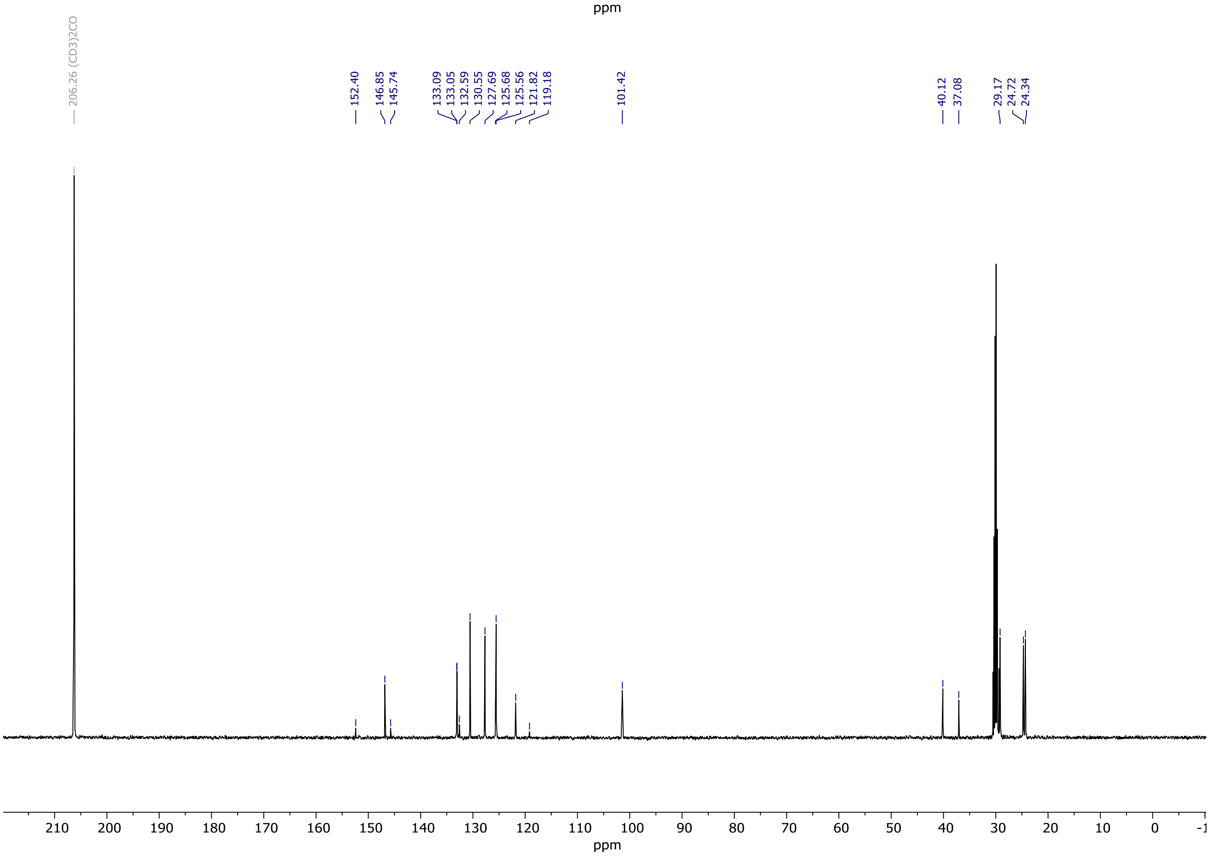

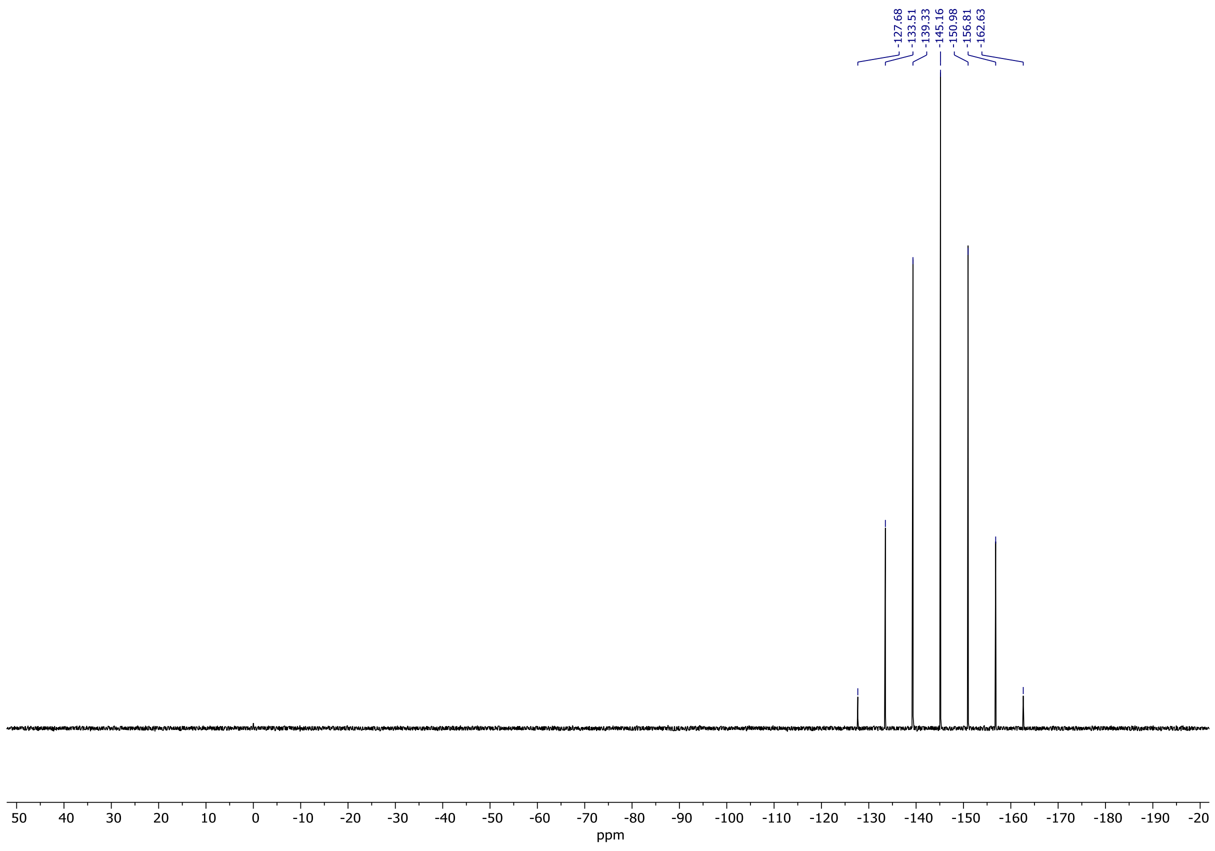
-127.68
-133.51
-139.33
-145.16
-150.98
-156.81
-162.63
50 40 30 20 10 0 -10 -20 -30 -40 -50 -60 -70 -80 -90 -100 -110 -120 -130 -140 -150 -160 -170 -180 -190 -20
ppm

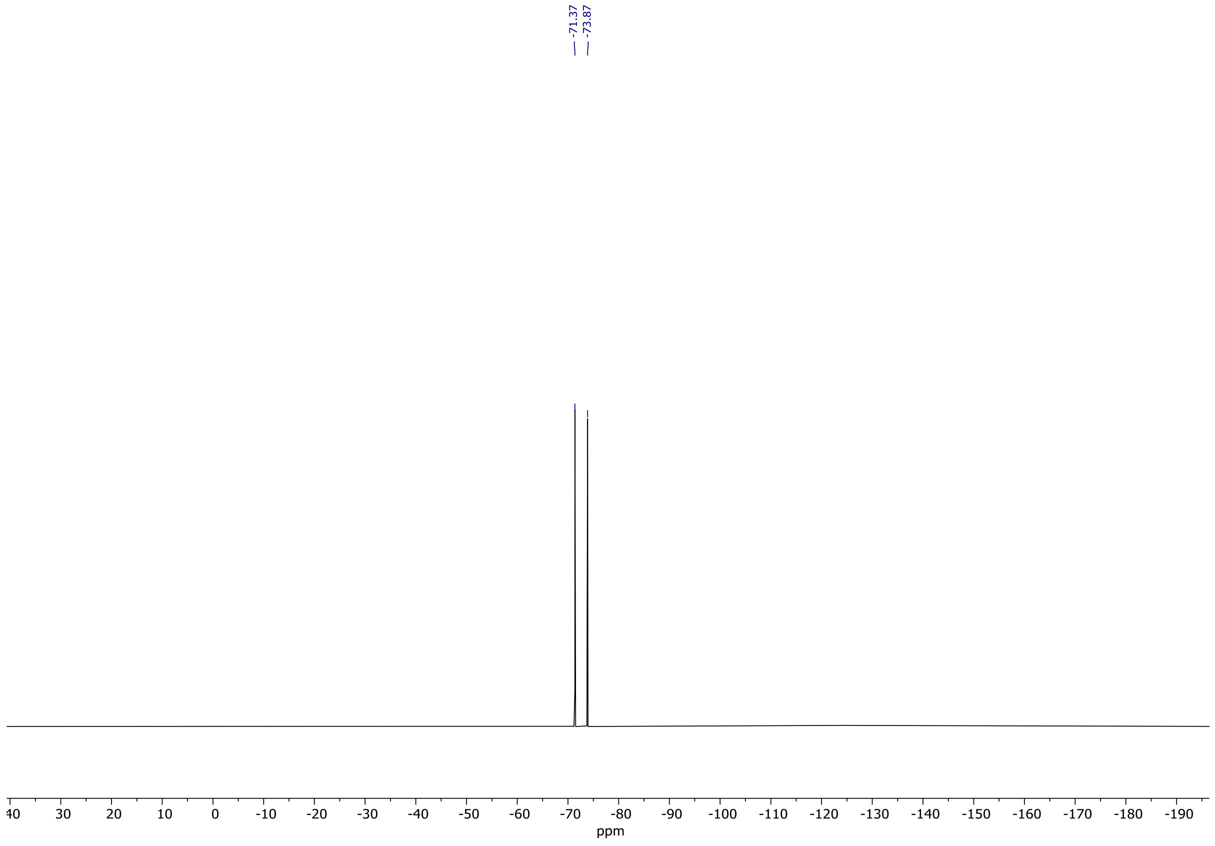
-71.37
-73.87
40 30 20 10 0 -10 -20 -30 -40 -50 -60 -70 -80 -90 -100 -110 -120 -130 -140 -150 -160 -170 -180 -190
ppm

6-(2,6-Diisopropylphenyl)-2-(4-methoxyphenyl)-3-(*N*-methylmethylsulfonamido)imidazo[5,1-*b*]oxazol-6-ium hexafluorophosphate(V) (9b) in $CDCl_3$ ^{1}H, ^{13}C UDEFT, $^{31}P\{^{1}H\}$ and $^{19}F\{^{1}H\}$ NMR

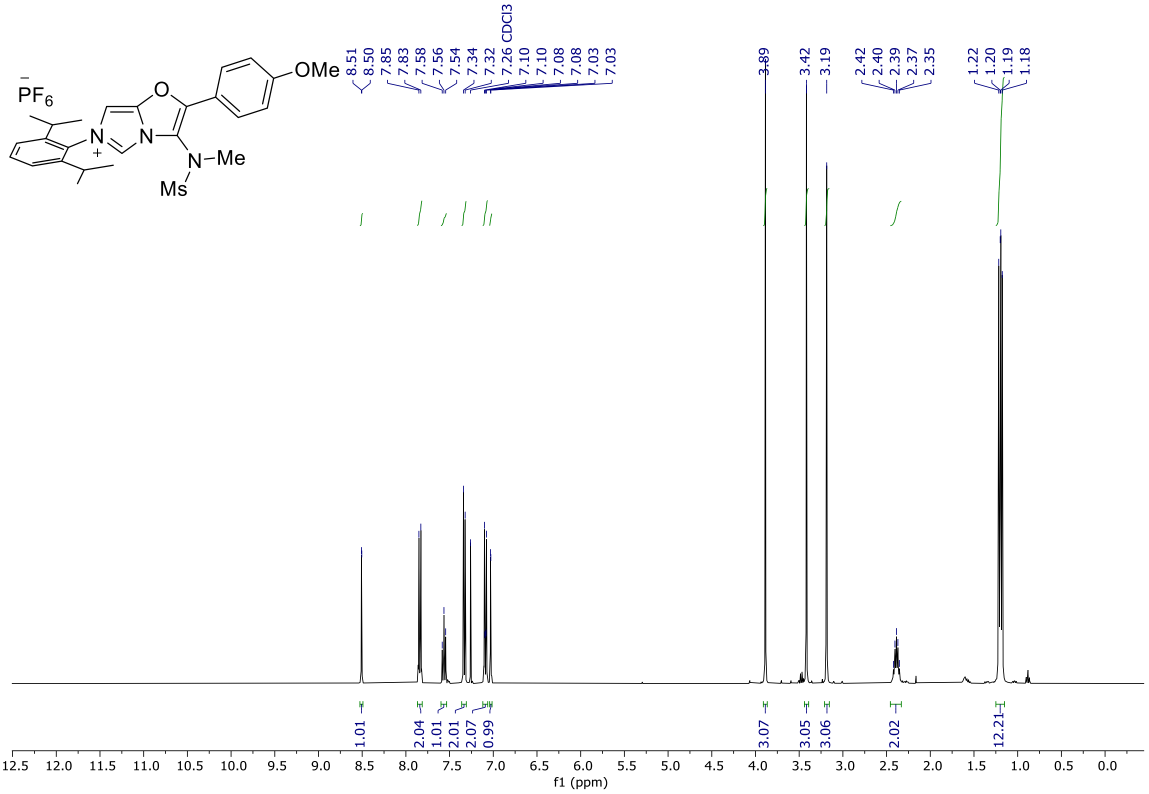

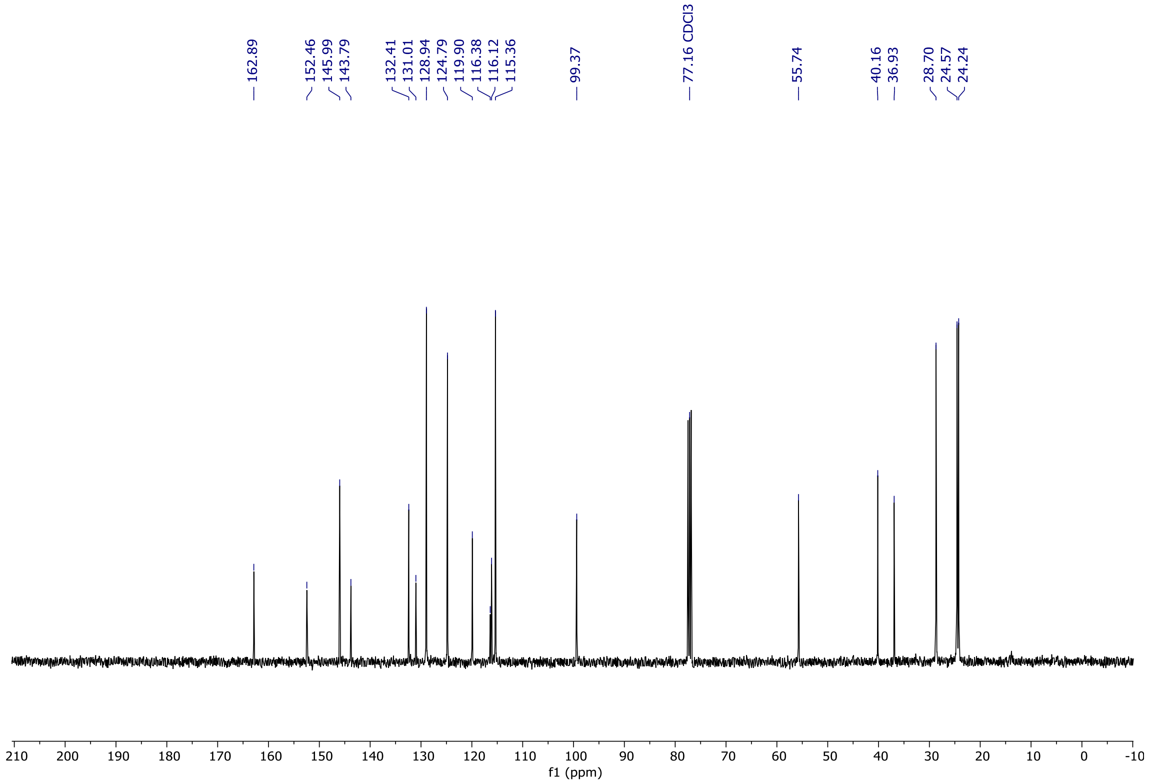

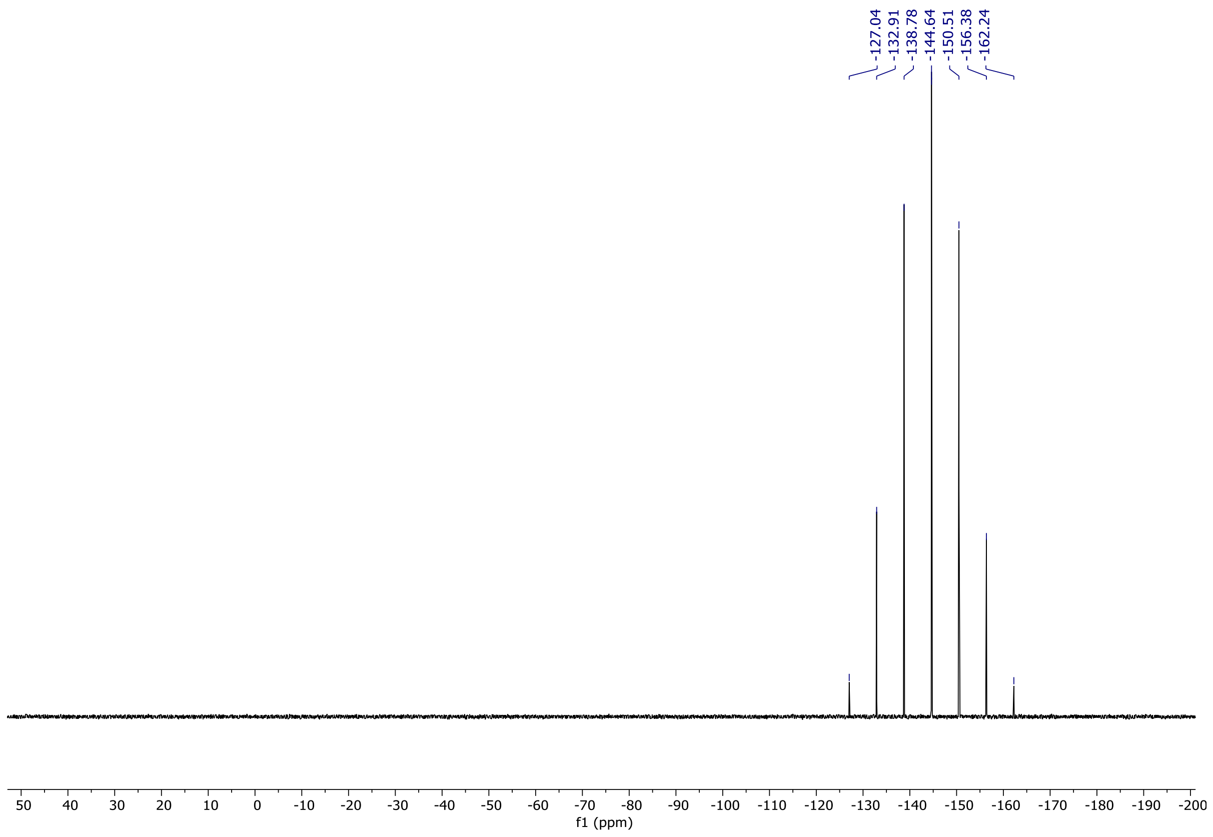
-127.04
-132.91
-138.78
-144.64
-150.51
-156.38
-162.24
50 40 30 20 10 0 -10 -20 -30 -40 -50 -60 -70 -80 -90 -100 -110 -120 -130 -140 -150 -160 -170 -180 -190 -200
f1 (ppm)

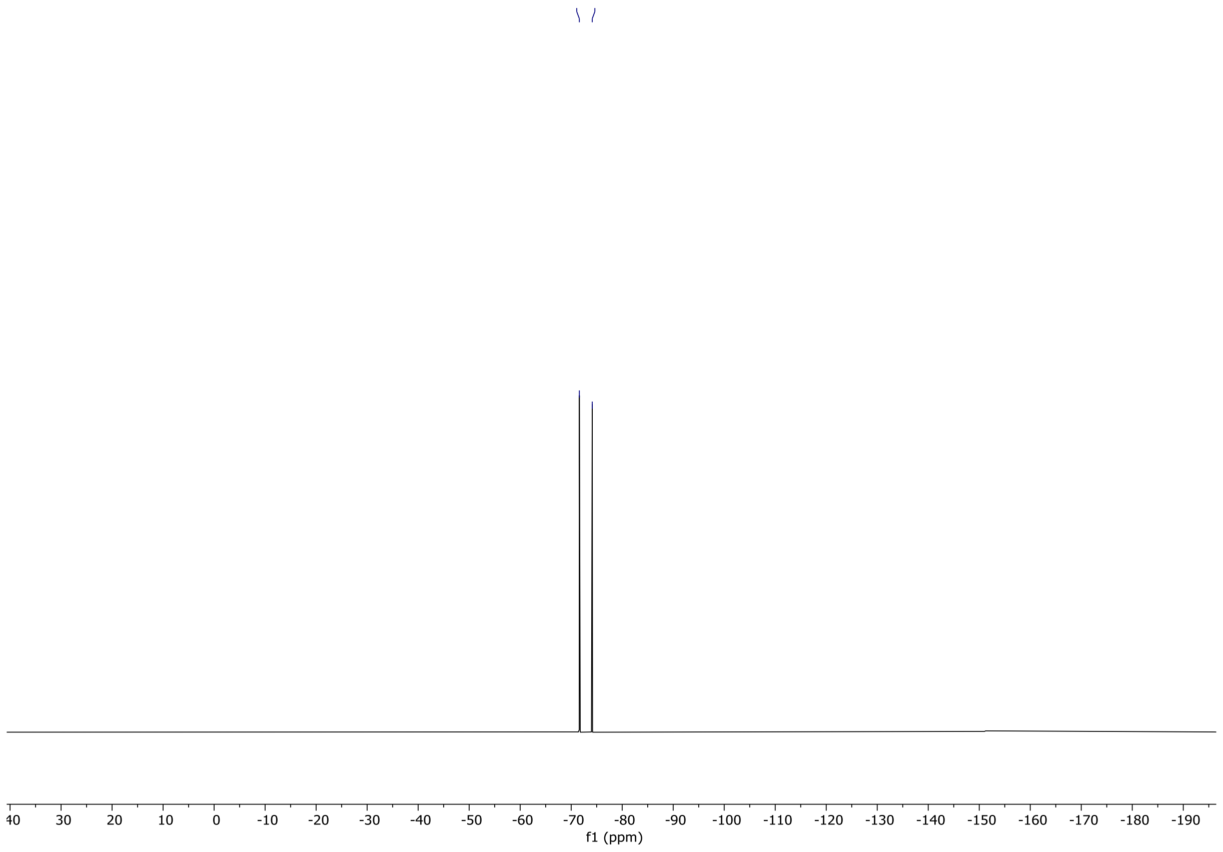
40 30 20 10 0 -10 -20 -30 -40 -50 -60 -70 -80 -90 -100 -110 -120 -130 -140 -150 -160 -170 -180 -190
f1 (ppm)

(2,2-Dimethoxyacetyl)(pyridin-1-ium-1-yl)amide (4) in $CDCl_3$ ^1H and ^{13}C PENDANT NMR

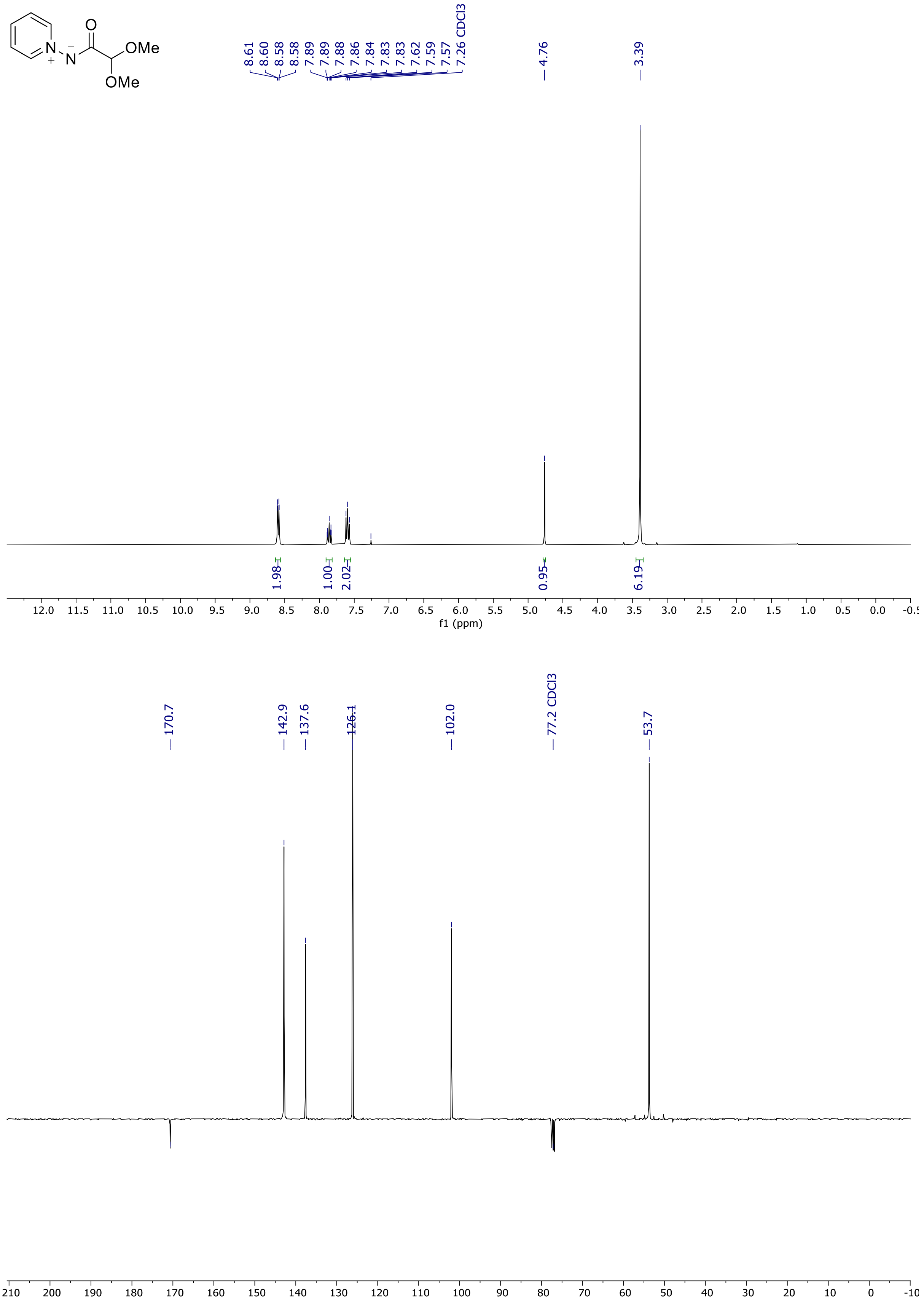

_N_-(2-(Dimethoxymethyl)-5-phenyloxazol-4-yl)-_N_-methylmethanesulfonamide (5) in $CDCl_3$ ^1H and ^{13}C PENDANT NMR

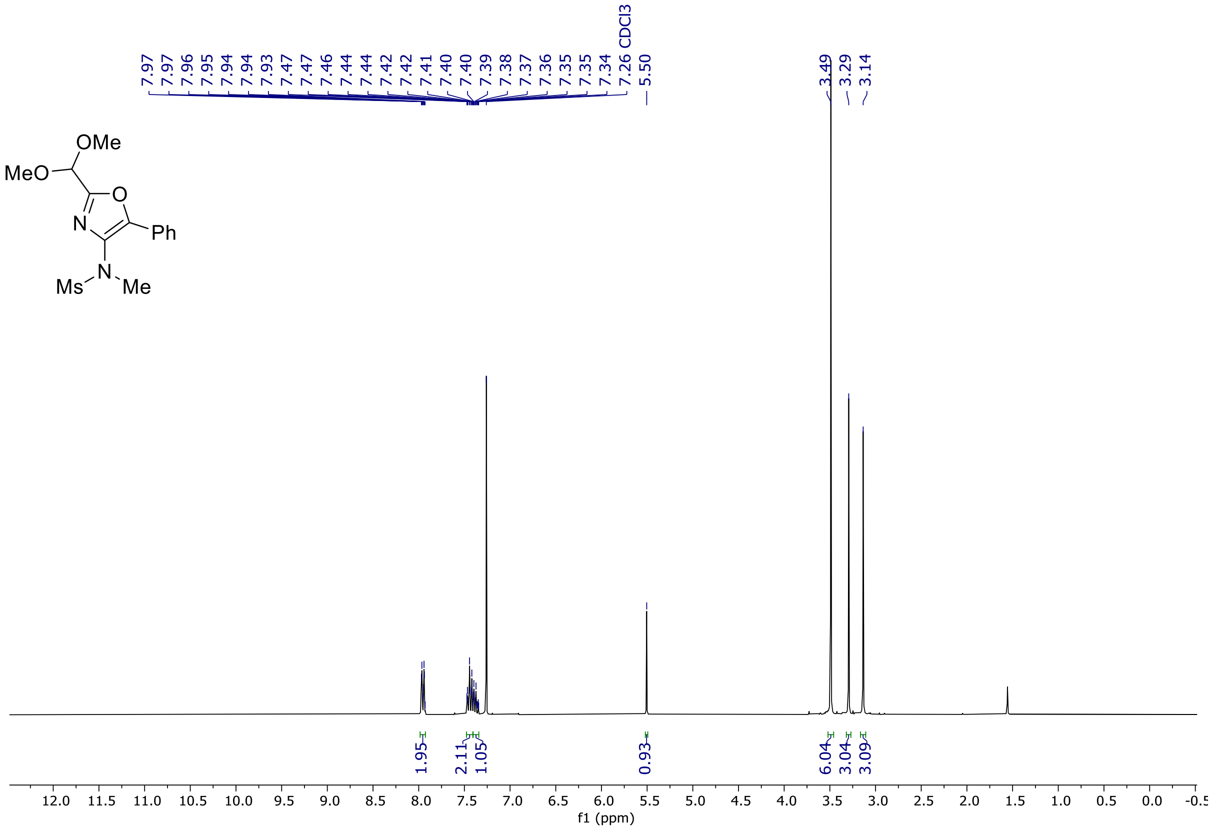

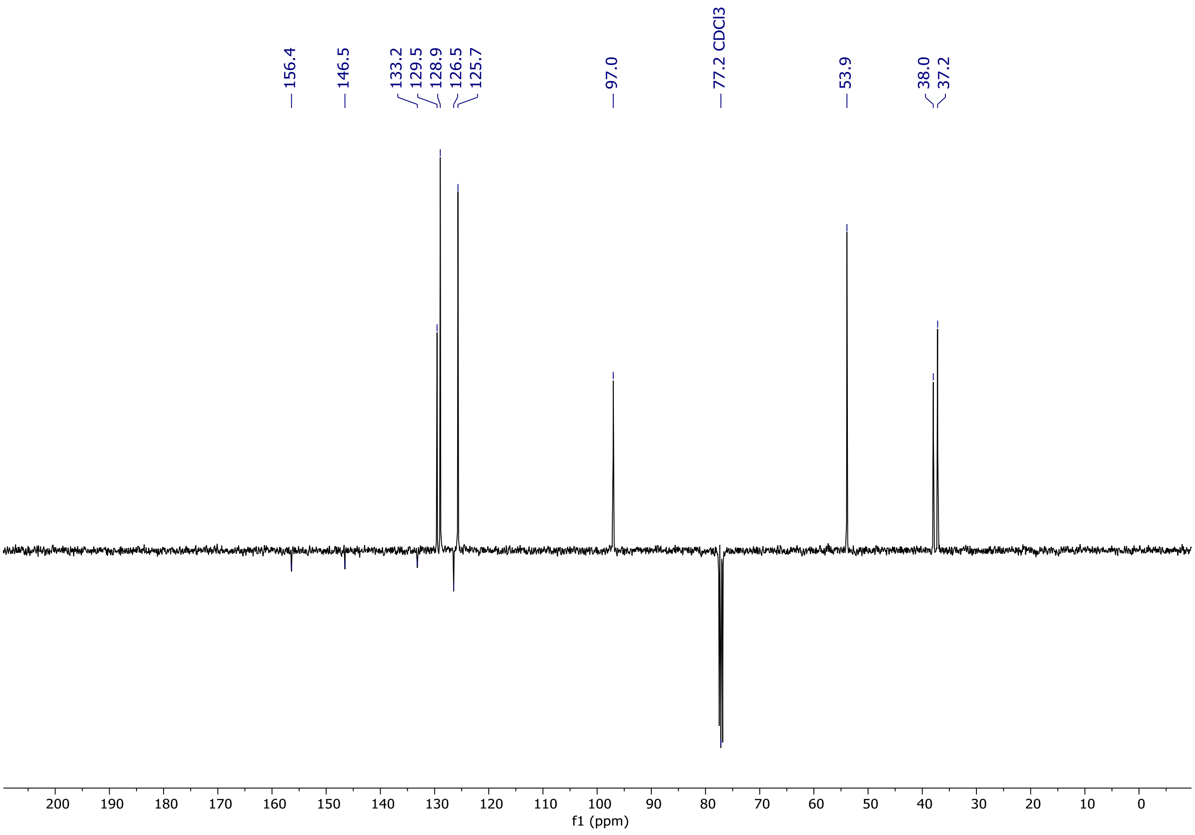

(*E*)-*N*-(2-(((2,6-Diisopropylphenyl)imino)methyl)-5-phenyloxazol-4-yl)-*N*-methylmethanesulfonamide (6) in $CDCl_3$ ^{1}H and ^{13}C PENDANT NMR

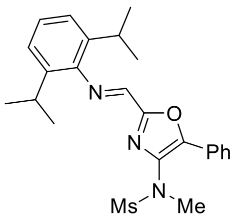

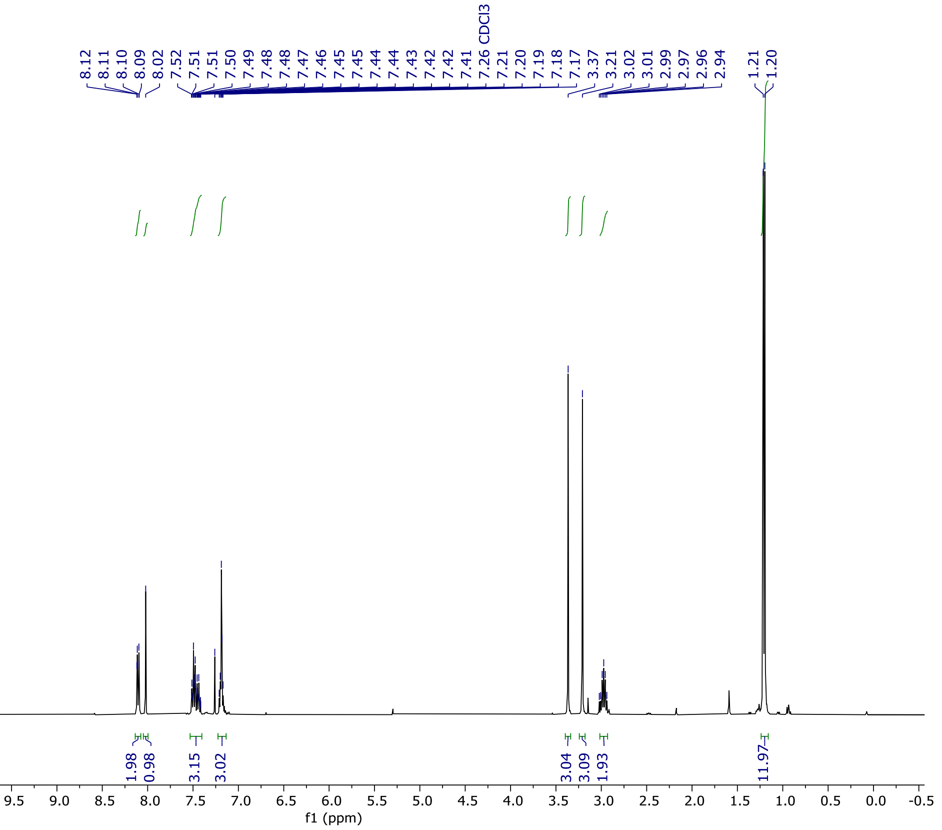

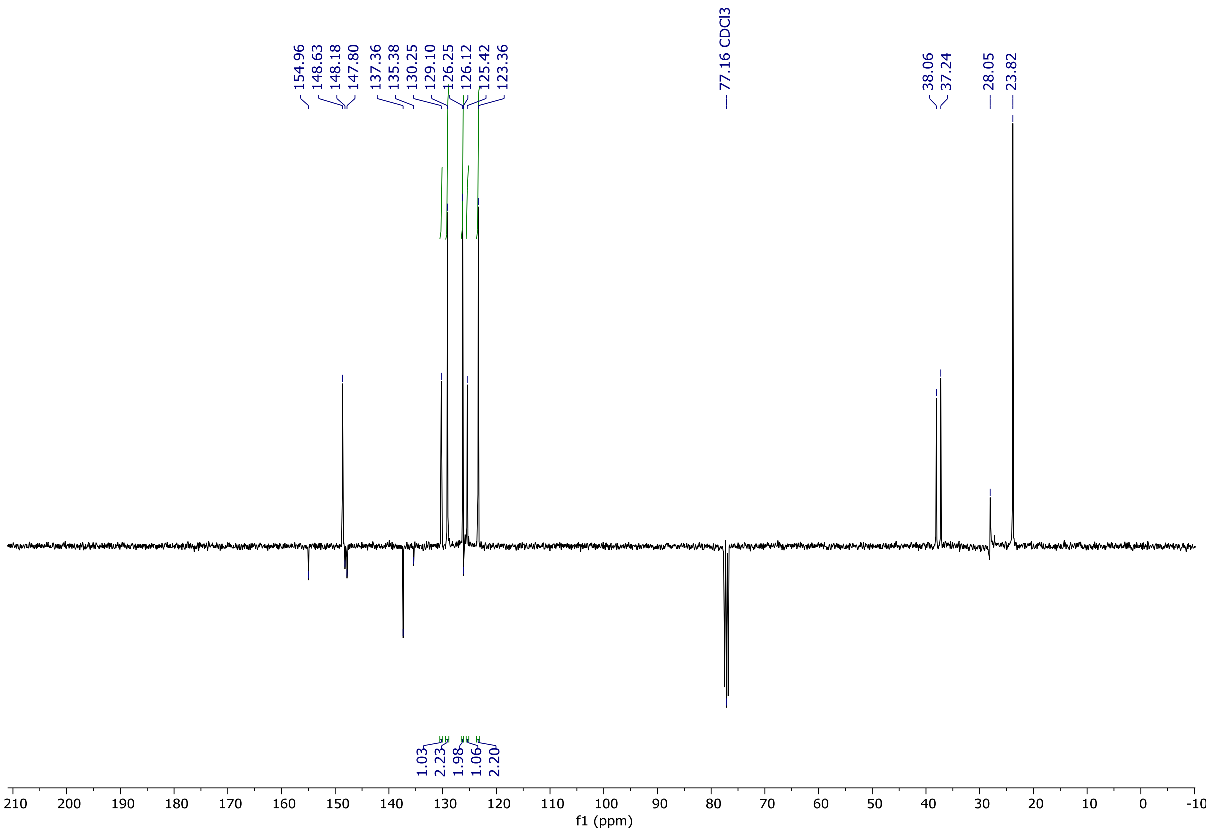

Methyl (2,6-diisopropylphenyl)glycinate (11) in $CDCl_3$ ^1H and ^{13}C PENDANT NMR

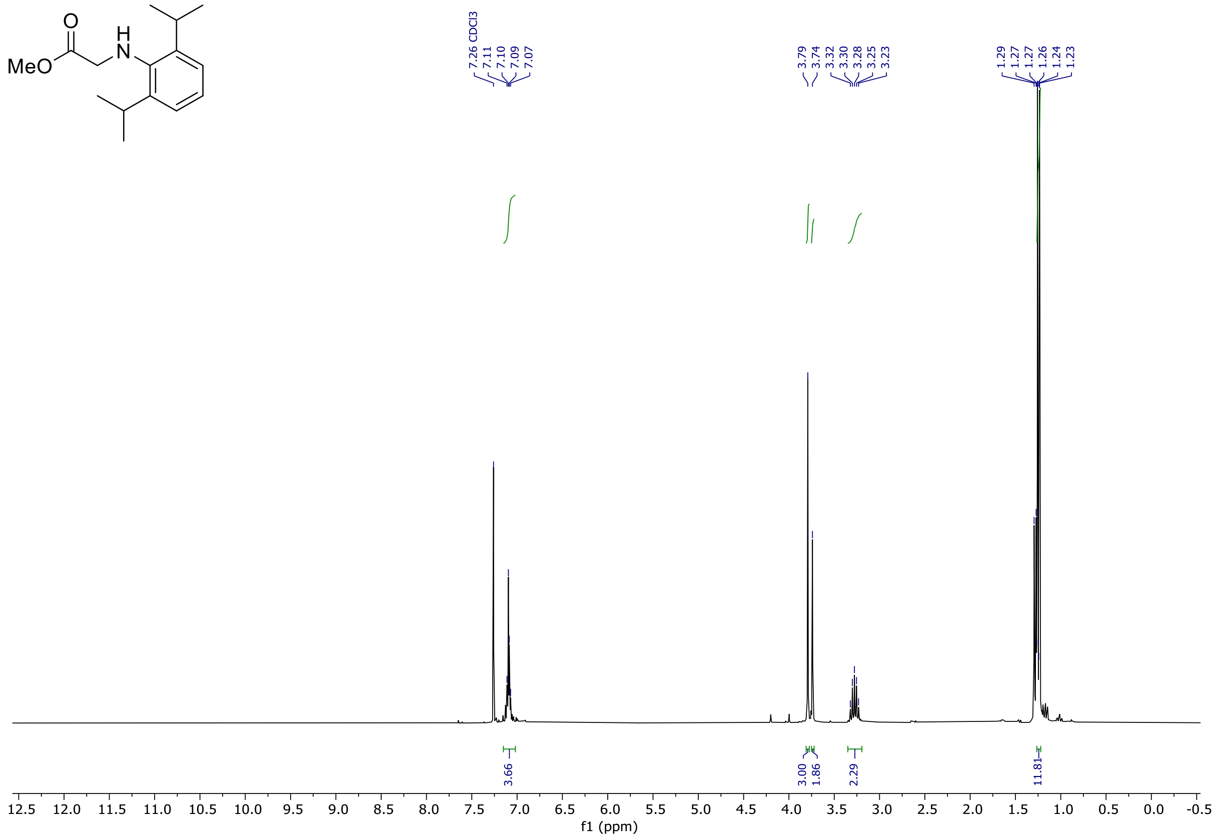

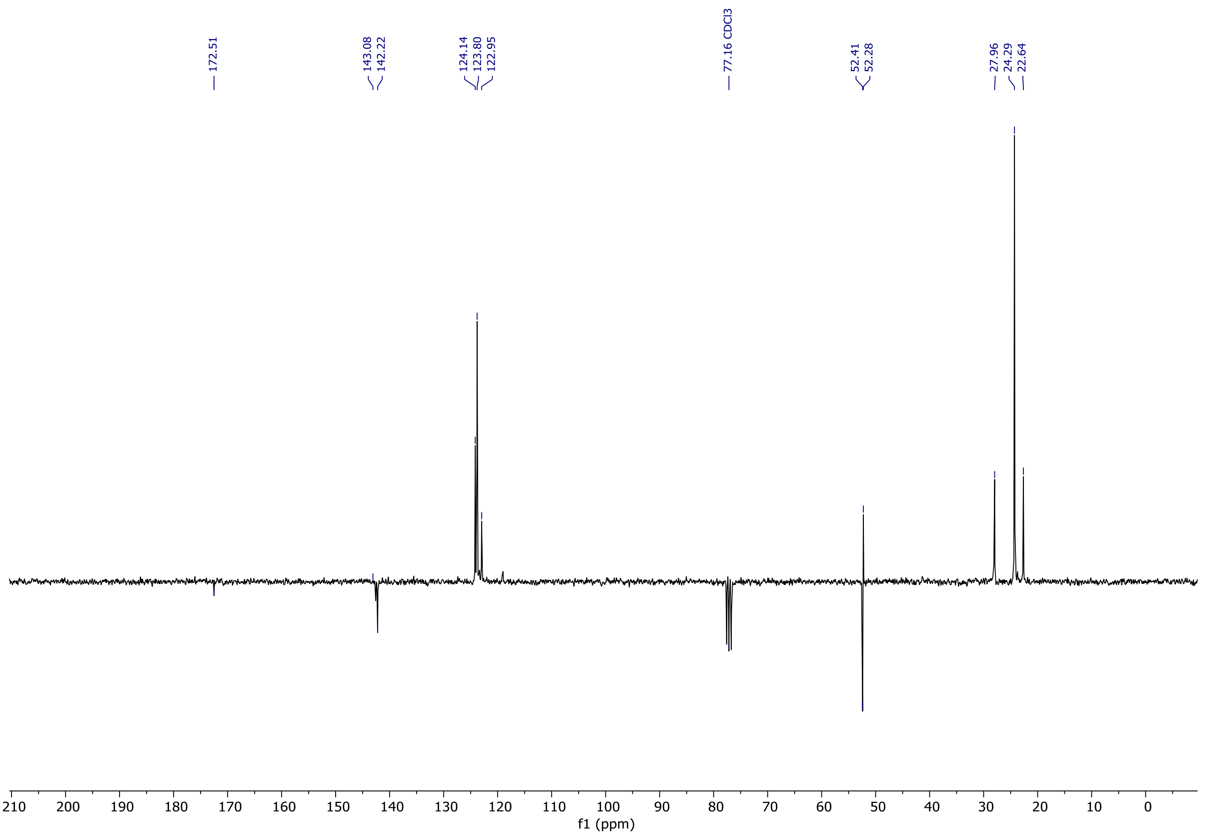

Methyl *N*-(2,6-diisopropylphenyl)-*N*-formylglycinate (12) in $CDCl_3$ ^{1}H and ^{13}C PENDANT NMR

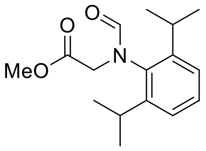

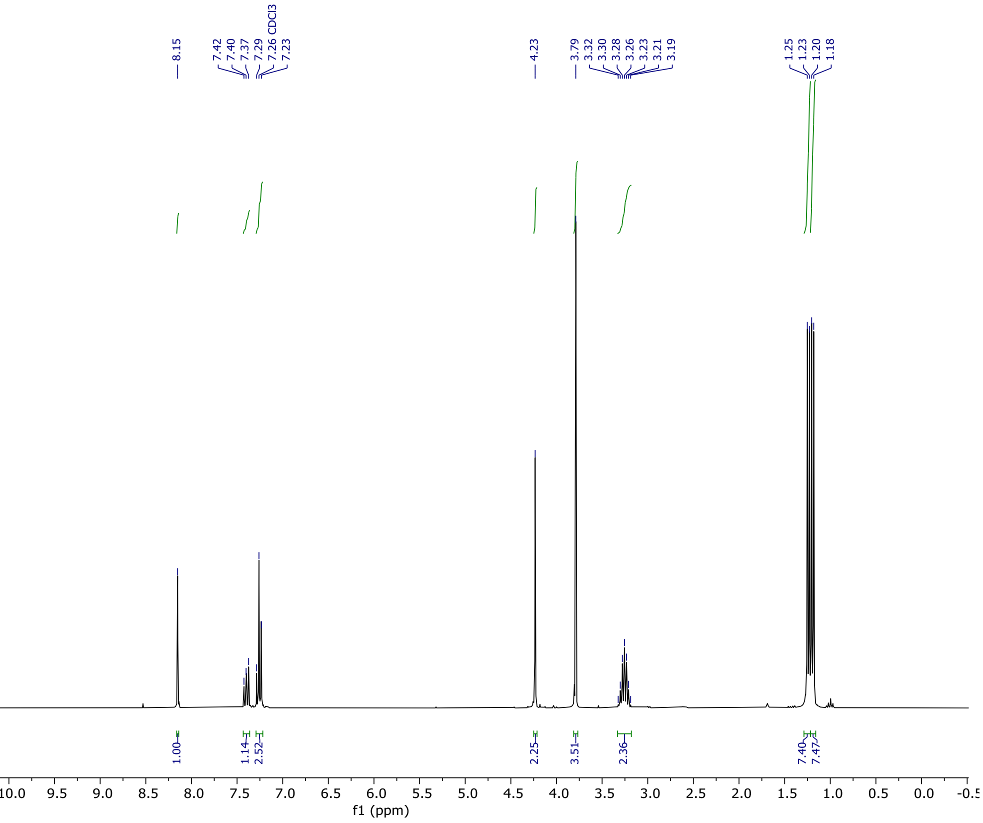

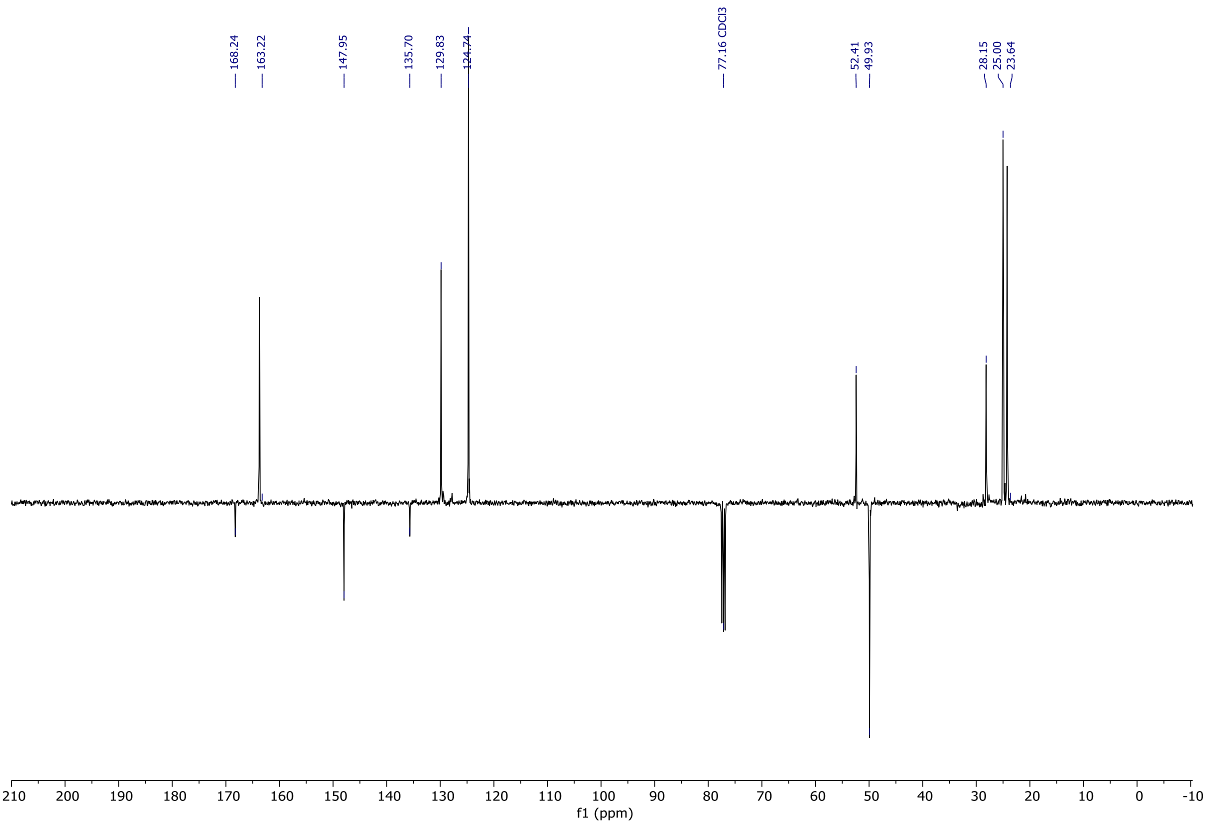

AImOxAuCl (13) in $CDCl_3$ ^1H and ^{13}C UDEFT NMR

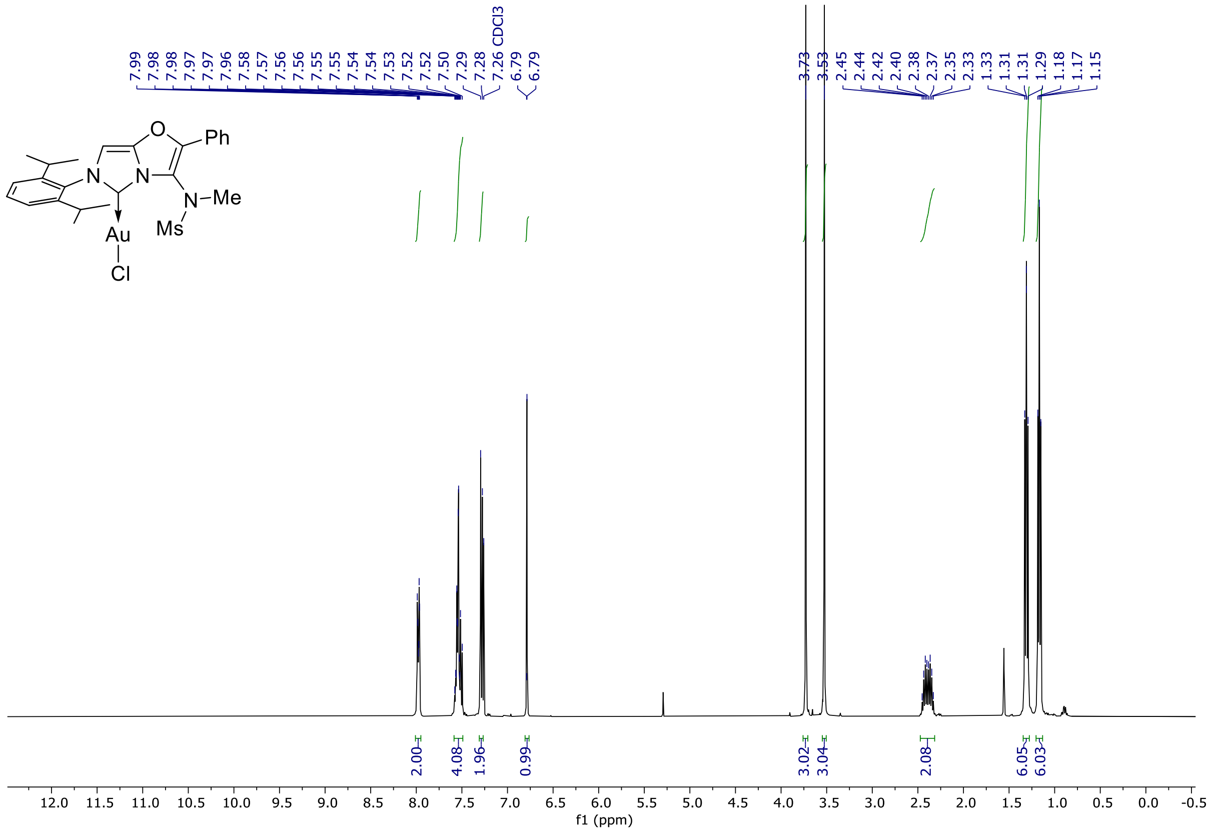

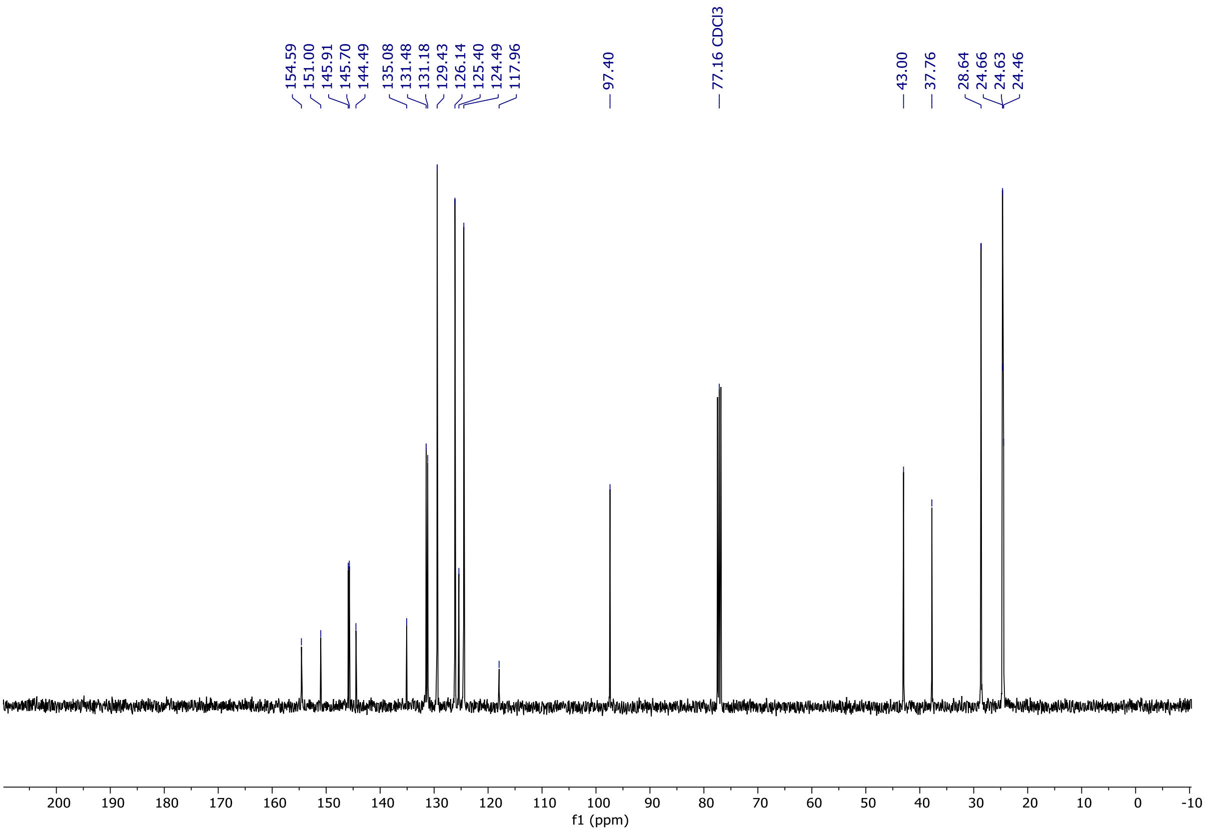

AImOxCuCl (14) in $CDCl_3$ ^1H and ^{13}C UDEFT NMR

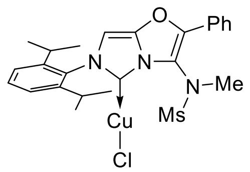

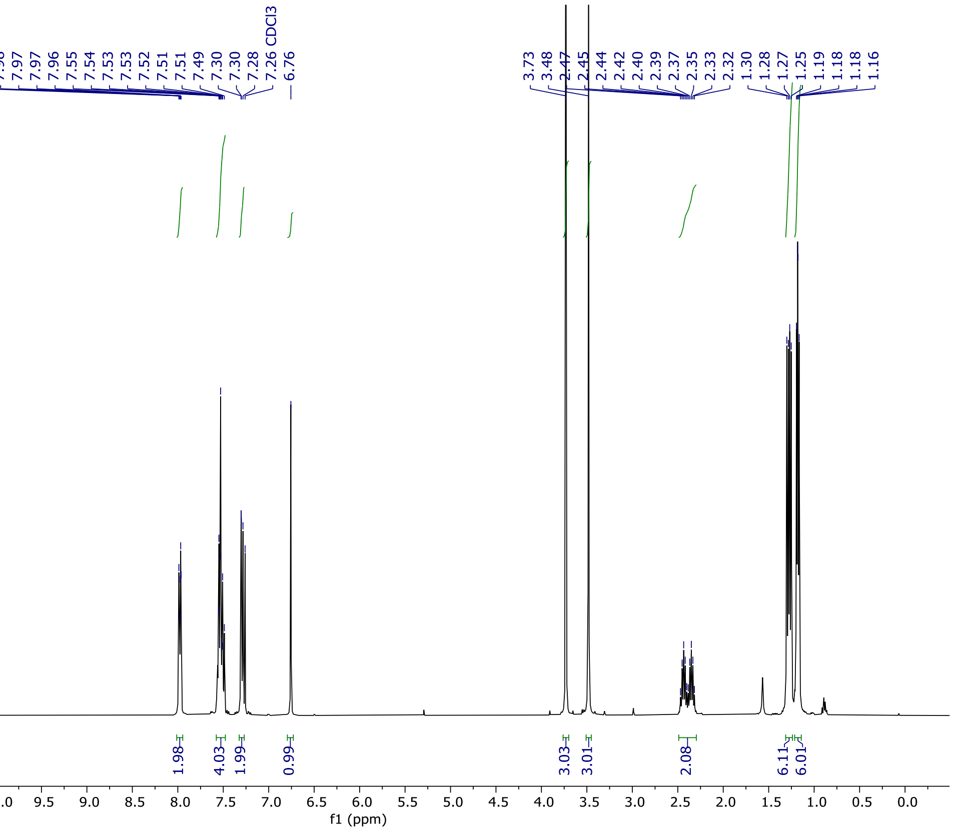

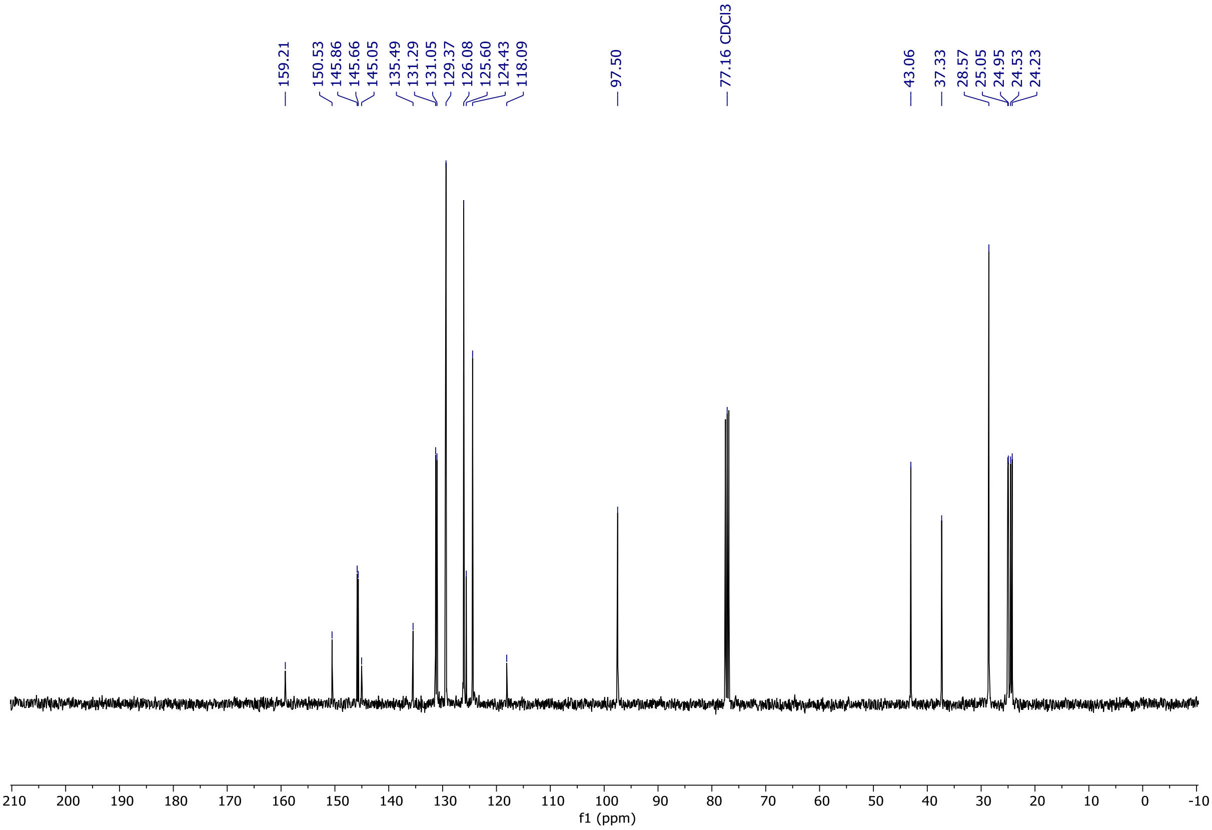

(9a)Ir(CO)$_2$Cl (15a) (Diastereomeric mixture) in $CDCl_3$ ^{1}H and ^{13}C UDEFT NMR

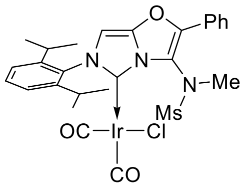

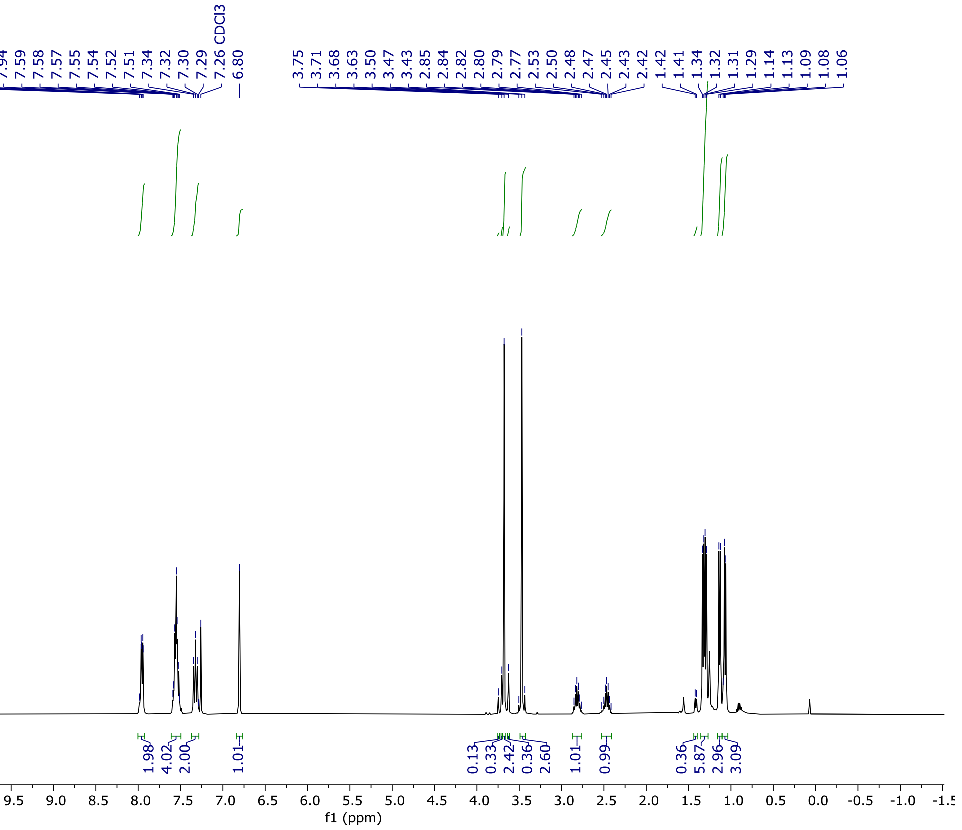

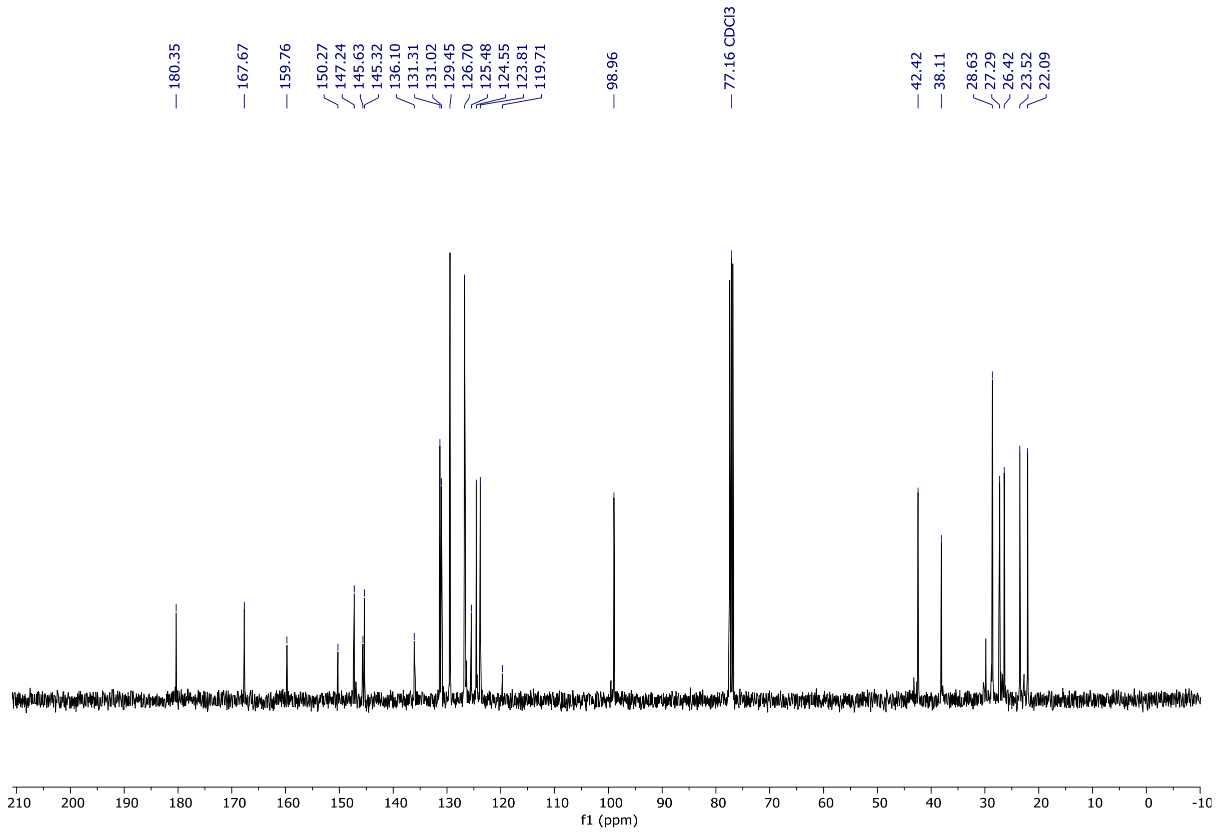

(9b)Ir(CO)$_2$Cl (15b) (Diastereomeric mixture) in CDCl$_3$ ^{1}H and ^{13}C UDEFT NMR

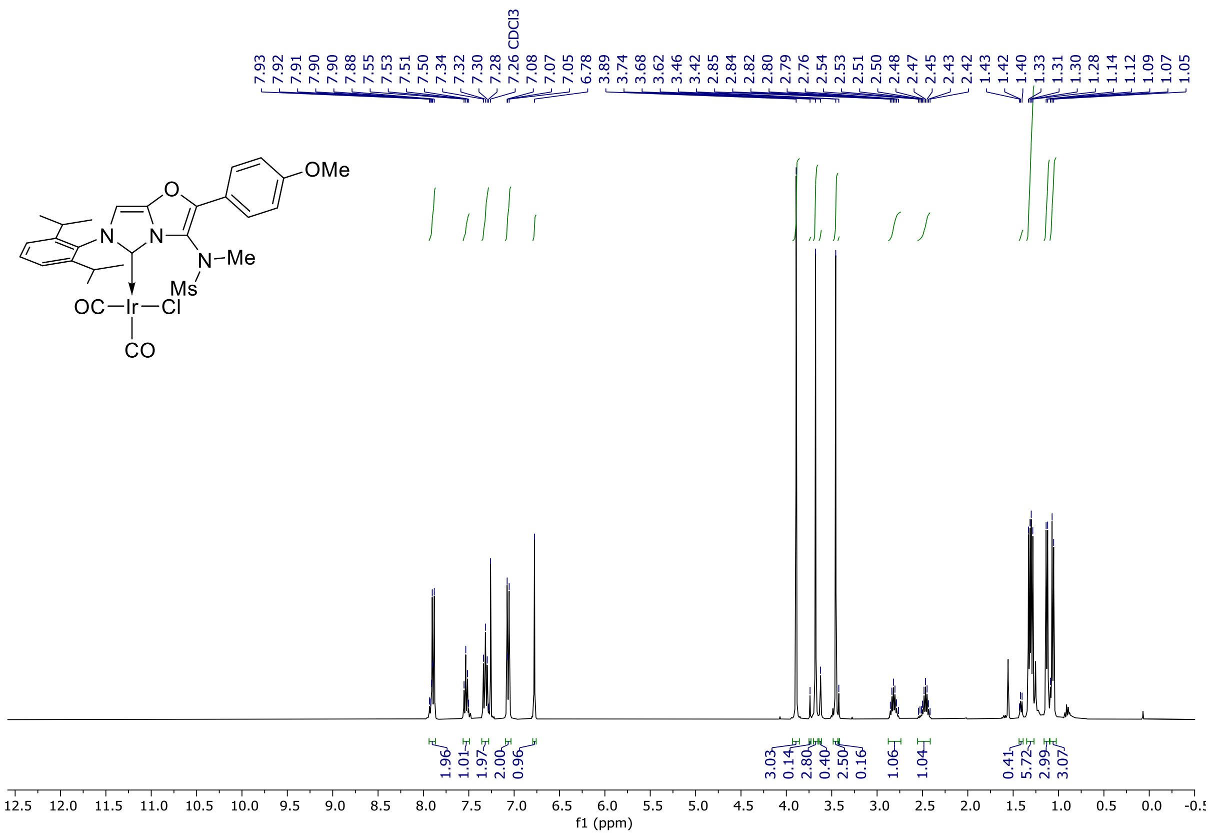

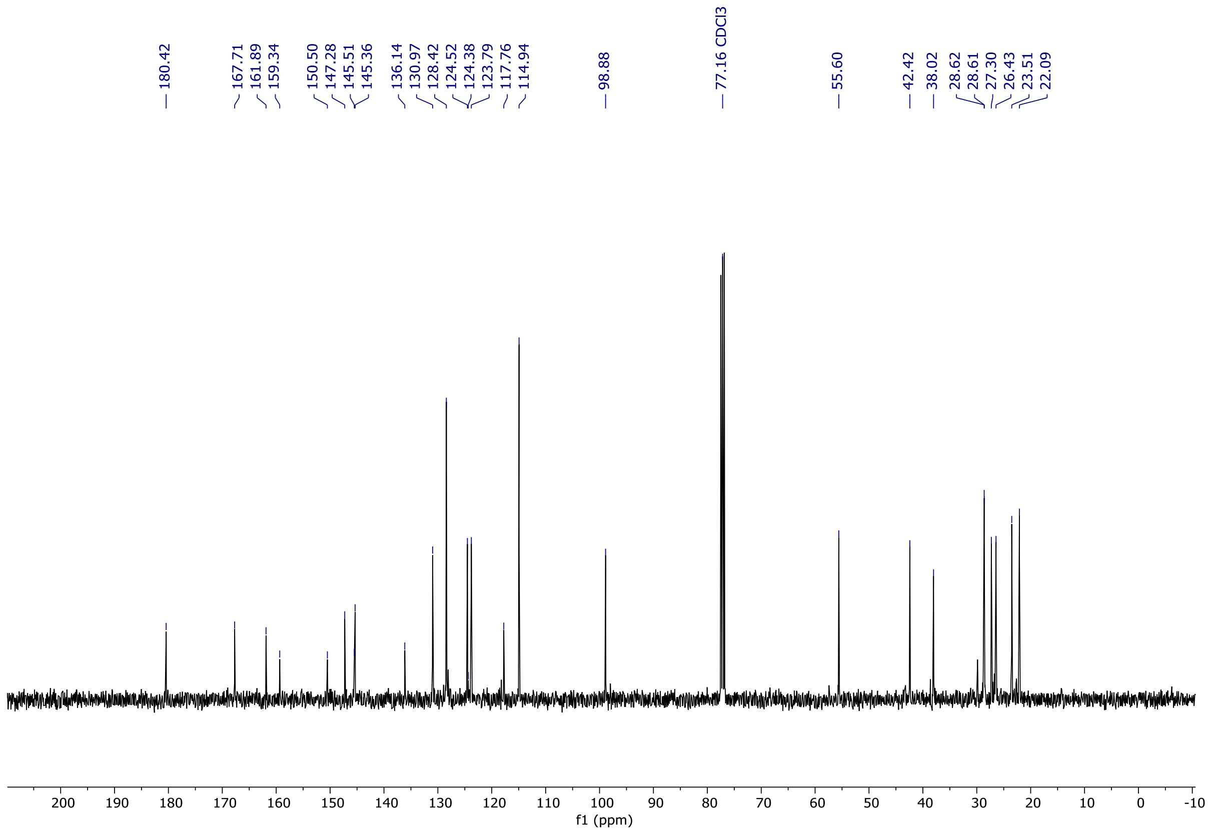

Crystallographic data

The dataset for **13** was measured on an Agilent SuperNova diffractometer using an Atlas detector. The data collection was driven and processed and an absorption correction was applied using CrysAlisPro [22]. The dataset for **14** was measured by the UK National Crystallography Service on a Rigaku Saturn724+ diffractometer equipped with a rotating anode molybdenum source and a AFC12 goniometer. The data collection was driven and processed and an absorption correction was applied using CrystalClear-SM Expert 3.1 [23]. Both structures were solved using ShelXS [24] and refined by a full-matrix least-squares procedure on F^2 in ShelXL [25]. Figures and reports were produced using OLEX2 [26]. All non-hydrogen atoms were refined with anisotropic displacement parameters. All hydrogen atoms in both structures and the deuterium atom in **14** were fixed as riding models and the isotropic thermal parameters (U_{iso}) were based on the U_{eq} of the parent atoms.

Structure **14** contains a molecule of deuterated chloroform.

CCDC 2310256-2310257 contain the supplementary crystallographic data for this paper. These data can be obtained free of charge from The Cambridge Crystallographic Data Centre *via* https://www.ccdc.cam.ac.uk/data_request/cif.

Crystal data for **13**: $C_{25}H_{29}AuClN_3O_3S$ (M =683.99 g/mol): monoclinic, space group $P2_1/n$ (no. 14), a = 9.9647(3) Å, b = 20.0858(10) Å, c = 12.9728(4) Å, β = 100.759(3)°, V = 2550.85(16) Å^3, Z = 4, T = 100.00(10) K, μ(Mo Kα) = 5.986 mm^{-1}, $Dcalc$ = 1.781 g/cm^3, 13660 reflections measured (4.628° ≤ 2Θ ≤ 52.738°), 5212 unique (R_{int} = 0.0310, R_{sigma} = 0.0428) which were used in all calculations. The final R_1 was 0.0357 (I > 2σ(I)) and wR_2 was 0.0721 (all data).

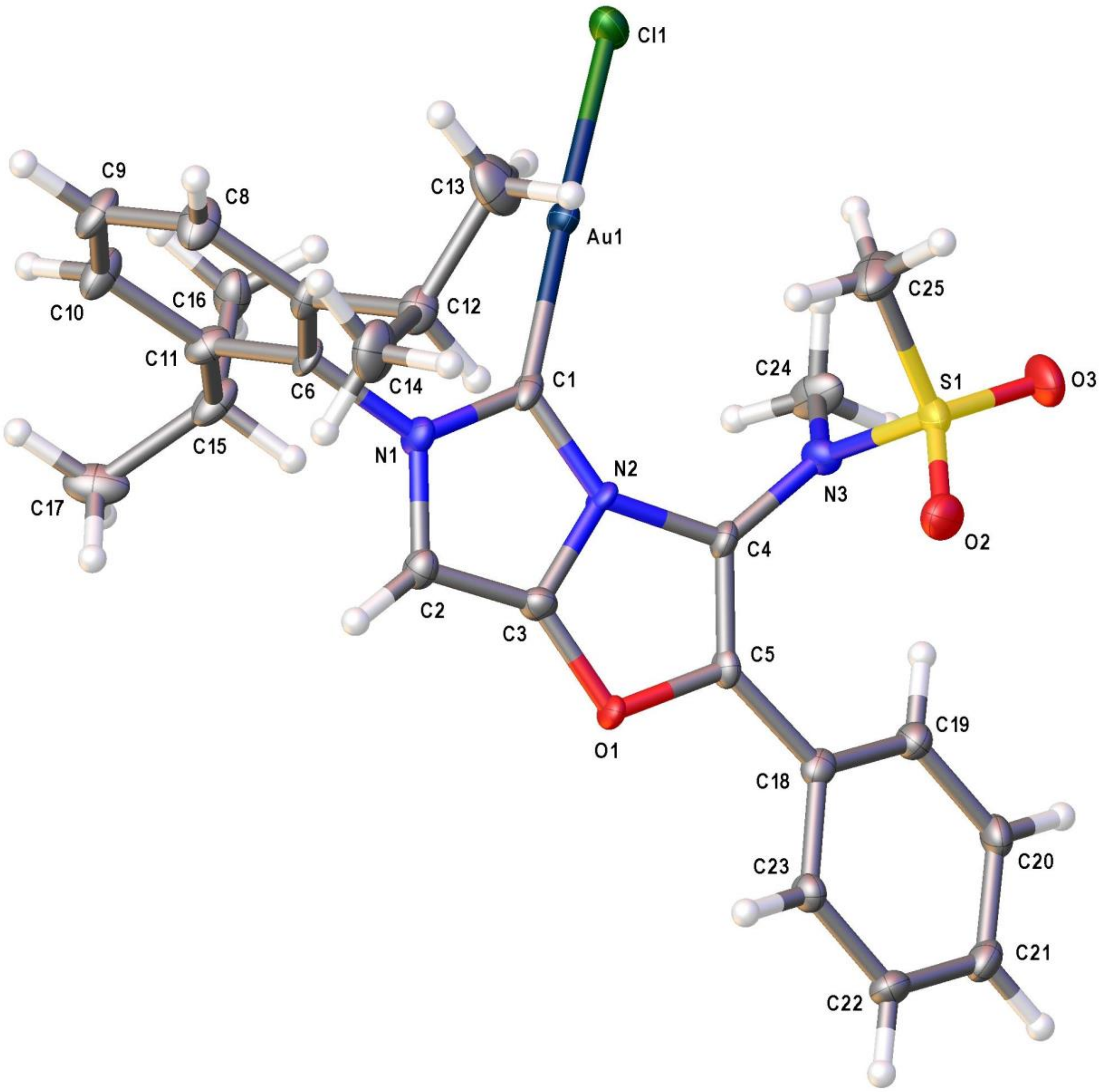

Figure S1 *Crystal structure of **13** with ellipsoids drawn at the 50 % probability level.*

Crystal data for **14**: $C_{26}H_{29}DN_3O_3SCl_4Cu$ (M =670.97 g/mol): monoclinic, space group $P2_1/n$ (no. 14), a = 16.9828(3) Å, b = 10.1478(2) Å, c = 17.4535(12) Å, β = 95.615(7)°, V = 2993.5(2) Å^3, Z = 4, T = 100(2) K, μ(MoKα) = 1.189 mm^{-1}, $Dcalc$ = 1.489 g/cm^3, 39227 reflections measured (6.174° ≤ 2Θ ≤ 54.96°), 6850 unique (R_{int} = 0.0259, R_{sigma} = 0.0168) which were used in all calculations. The final R_1 was 0.0319 (I > 2σ(I)) and wR_2 was 0.0820 (all data).

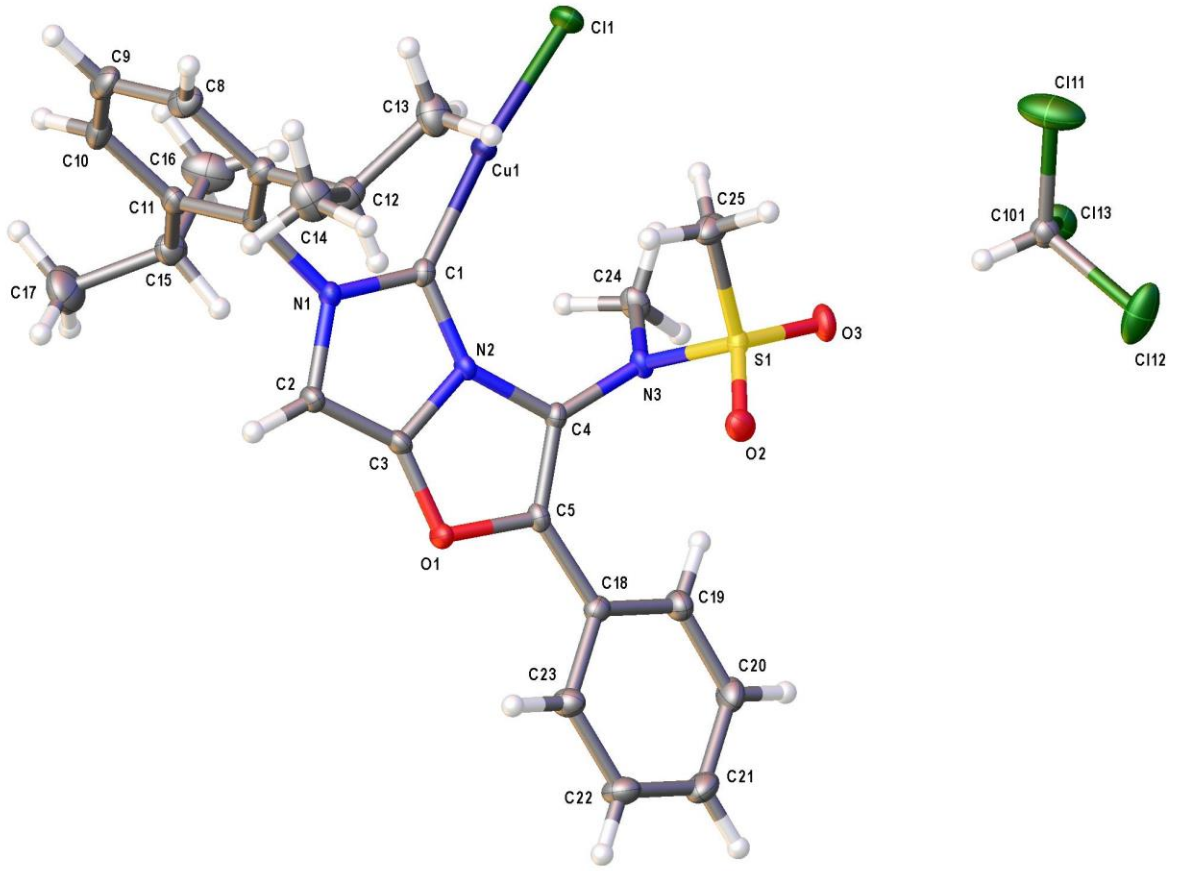

Figure S2 *Crystal structure of **14** with ellipsoids drawn at the 50% probability level.*

References

1. Johnson, D. C.; Widlanski, T. S. *J. Org. Chem.* **2003**, *68*, 5300-5309.
2. Rao, M. L.; Jadhav, D. N.; Dasgupta, P. *Org. Lett.* **2010**, *12*, 2048-2051.
3. Araki, Y.; Kobayashi, K.; Yonemoto, M.; Kondo, Y. *Org. Biomol. Chem.* **2011**, *9*, 78-80.
4. Zhang, X.; Zhang, Y.; Huang, J.; Hsung, R. P.; Kurtz, K. C. M.; Oppenheimer, J.; Petersen, M. E.; Sagamanova, I. K.; Shen, L.; Tracey, M. R. *J. Org. Chem.* **2006**, *71*, 4170-4177.
5. Dateer, R. B.; Shaibu, B. S.; Liu, R.-S. *Angew. Chem., Int. Ed.* **2012**, *51*, 113-117.
6. Mukherjee, A.; Dateer, R. B.; Chaudhuri, R.; Bhunia, S.; Karad, S. N.; Liu, R.-S. *J. Am. Chem. Soc.* **2011**, *133*, 15372-15375.
7. Coste, A.; Karthikeyan, G.; Couty, F.; Evano, G. *Angew. Chem., Int. Ed.* **2009**, *48*, 4381-4385.
8. Xie, J.; Huang, Z.-Z. *Angew. Chem. Int. Ed.* **2010**, *49*, 10181-10185.
9. Cameron, A. G.; Hewson, A. T. *J. Chem. Soc., Perkin Trans. 1* **1983**, 2979-2982.
10. Alcarazo, M.; Roseblade, S. J.; Cowley, A. R.; Fernández, R.; Brown, J. M.; Lassaletta, J. M. *J. Am. Chem. Soc.* **2005**, *127*, 3290-3291.
11. Collado, A.; Gómez-Suárez, A.; Martin, A. R.; Slawin, A. M. Z.; Nolan, S. P. *Chem. Commun.* **2013**, *49*, 5541-5543.
12. Walkinshaw, A. J.; Xu, W.; Suero, M. G.; Gaunt, M. J. *J. Am. Chem. Soc.* **2013**, *135*, 12532-12535.
13. Cecchini, C.; Lanzi, M.; Cera, G.; Malacria, M.; Maestri, G. *Synthesis* **2019**, *51*, 1216-1224.
14. Nieto-Oberhuber, C.; López, S.; Echavarren, A. M. *J. Am. Chem. Soc.* **2005**, *127*, 6178-6179.
15. Martí, À.; Montesinos-Magraner, M.; Echavarren, A. M.; Franchino, A. *Eur. J. Org. Chem.* **2022**, *2022*, e202200518.
16. Marion, N.; Ramón, R. S.; Nolan, S. P. *J. Am. Chem. Soc.* **2009**, *131*, 448-449.
17. Zhao, C.; Jia, X.; Wang, X.; Gong, H. *J. Am. Chem. Soc.* **2014**, *136*, 17645-17651.
18. Hsieh, J.-C.; Chen, Y.-C.; Cheng, A.-Y.; Tseng, H.-C. *Org. Lett.* **2012**, *14*, 1282-1285.
19. Tang, Y.; Benaissa, I.; Huynh, M.; Vendier, L.; Lugan, N.; Bastin, S.; Belmont, P.; César, V.; Michelet, V. *Angew. Chem. Int. Ed.* **2019**, *58*, 7977-7981.
20. Vanitcha, A.; Damelincourt, C.; Gontard, G.; Vanthuyne, N.; Mouriès-Mansuy, V.; Fensterbank, L. *Chem. Commun.* **2016**, *52*, 6785-6788.
21. Aillard, P.; Dova, D.; Magné, V.; Retailleau, P.; Cauteruccio, S.; Licandro, E.; Voituriez, A.; Marinetti, A. *Chem. Commun.* **2016**, *52*, 10984-10987.
22. CrysAlisPro, Rigaku Oxford Diffraction, 2015
23. CrystalClear-SM Expert 3.1 b18, Rigaku Oxford Diffraction, 2012.
24. Sheldrick, G. *Acta. Cryst.* **2008**, *64*, 112-122.

25. Sheldrick, G. M. *Acta. Cryst.* **2015**, *C71*, 3-8.

26. Dolomanov, O. V.; Bourhis, L. J.; Gildea, R. J.; Howard, J. A. K.; Puschmann, H. *J. Appl. Crystallogr.* **2009**, *42*, 339-341.
